# Probing $\textit{CP}$-violating Higgs and gauge-boson couplings in the Standard Model effective field theory

**DOI:** 10.1140/epjc/s10052-017-5226-6

**Published:** 2017-10-12

**Authors:** Felipe Ferreira, Benjamin Fuks, Verónica Sanz, Dipan Sengupta

**Affiliations:** 1 Department of Physics and Astronomy, University of Sussex, Brighton, BN1 9QH UK; 2 Departamento de Física, Universidade Federal da Paraíba, Caixa Postal 5008, João Pessoa, Paraíba 58051-970 Brazil; 3 Sorbonne Universités, Université Pierre et Marie Curie (Paris 06), UMR 7589, LPTHE, 75005 Paris, France; 4 CNRS, UMR 7589, LPTHE, 75005 Paris, France; 5 Institut Universitaire de France, 103 boulevard Saint-Michel, 75005 Paris, France; 6 Laboratoire de Physique Subatomique et de Cosmologie, Université Grenoble-Alpes, CNRS/IN2P3, Avenue des Martyrs 53, 38026 Grenoble, France; 7 Department of Physics and Astronomy, Michigan State University, East Lansing, USA

## Abstract

We study the phenomenological consequences of several *CP*-violating structures that could arise in the Standard Model effective field theory framework. Focusing on operators involving electroweak gauge and/or Higgs bosons, we derive constraints originating from Run I LHC data. We then study the capabilities of the present and future LHC runs at higher energies to further probe associated *CP*-violating phenomena and we demonstrate how differential information can play a key role. We consider both traditional four-lepton probes of *CP*-violation in the Higgs sector and novel new physics handles based on varied angular and non-angular observables.

## Introduction

While the discovery of the 125 GeV Higgs boson [1, 2] has been an emphatic triumph of the first run of the LHC, questions about the true nature of the new boson still persist. The measured properties of the Higgs boson are so far consistent with the Standard Model predictions within the margins of the theoretical and experimental uncertainties [3], but current data still leaves enough room for deviations. As a consequence, one of the main topics of the next LHC runs consists of precisely measuring the Higgs-boson properties, i.e., its couplings to the Standard Model particles and its *CP* nature.

One of the simplest model-independent way of analyzing deviations from the Standard Model in the properties of the Higgs boson relies on the effective field theory (EFT) language. In this approach, all new physics contributions to the Standard Model are parameterized in terms of higher-dimensional operators, the corresponding Wilson coefficients encoding the dependence on the ultraviolet completion of the Standard Model being taken as free parameters. The EFT approach can be tested per se by investigating the correlations among the signatures expected both at the LHC and in low-energy experiments, which equivalently constrains the allowed range for the Wilson coefficients in the light of current data. Focusing on the possibly *CP*-violating nature of the Higgs-boson interactions, data is currently consistent with a *CP*-even hypothesis, like in the Standard Model. There, however, still exists a large fraction of the Wilson coefficient parameter space where the Higgs boson could exhibit *CP*-odd couplings to vector bosons and fermions. While this regions is mostly phenomenologically and experimentally unexplored, it remains important for model building considerations, as new sources of *CP* violation (CPV) are necessary to realize electroweak baryogenesis [4].

The impact of higher-dimensional operators modifying the way in which the Higgs boson interacts with the electroweak bosons has been extensively probed in the past. Most studies, however, assume that the new physics contributions to the Higgs-boson couplings feature a *CP*-even structure, in particular when existing constraints on the effective operators are evaluated [5–12]. In comparison, the investigation of the effects of the *CP*-odd Higgs-boson effective operators has been relatively sparse [13–19], although some experimental analyses are available, e.g. [20, 21]. As far as gauge interactions are concerned, CPV effects can be parameterized by six independent dimensions-six operators yielding novel interactions involving at least either three gauge and Higgs bosons, or gauge bosons only. The magnitude of the corresponding Wilson coefficients is in general constrained by electric dipole moments data and electroweak precision tests [16, 22–24], as well as by fits of Higgs coupling measurements at the LHC [25–29].

In the light of the amount of LHC data to be recorded in the following years, it is important to consider both options of CPV and *CP*-conserving new physics Higgs-boson interactions. The discrimination between these two kinds of effects is, however, only achievable once suitable observables allowing us to probe the *CP* nature of the Higgs couplings are considered. Pioneering work has followed this path and investigated handles that can be obtained from the study of asymmetries in specific observables [16, 30–33]. Effective scales $\varLambda$ that range up to 40 TeV have been found to be reachable with an LHC integrated luminosity of about $3000~\hbox {fb}^{-1}$, assuming ${\mathcal {O}}(1)$ Wilson coefficients.

The performed studies are, however, far from being exhaustive, both in terms of the considered set of differential distributions and the Higgs production and decay channels scrutinized. A significant number of other potential appealing options have indeed been left over, and could be used to unravel a potential *CP*-odd nature of the Higgs boson. In this paper, we focus on a dedicated set of observables that allows us to get a better handle on the CPV operators by studying several electroweak Higgs-boson production processes, as pointed out in the context of the LHC Higgs Cross Section Working Group [34]. We first consider dimensionful quantities for which the high-energy regime is automatically sensitive to the large momentum transfers induced by the EFT operators. We next consider angular observables that are naturally sensitive to the *CP*-violating nature of the considered operator. The complete quantitative analysis of this joint effect is left for future work.

The rest of the paper is organized as follows. In Sect. 2, we present the effective Lagrangian that we have used as a benchmark model, and we briefly discuss its possible connection to ultraviolet-complete extensions of the Standard Model in Sect. 3. In Sect. 4, we make use of the LHC Run I data to define the region of the Wilson coefficient parameter space that is relevant for the Run II studies that we have performed. Section 5 is dedicated to prospects arising from the use of total rates only, and Sect. 6 focuses on differential kinematic information. Our results are summarized and discussed in Sects. 7 and 8.

## Effective field theory framework

In the Standard Model EFT framework, all new physics effects are parameterized by means of higher-dimensional operators involving the Standard Model fields and assumed to stem from new phenomena occurring at a large energy scale $\varLambda$. Considering that the leading effects of physics beyond the Standard Model are described by operators of dimension six $\{ {\mathcal {O}}_i \}$, the Lagrangian modeling our theoretical framework is given by

1 $$\begin{aligned} {\mathcal {L}}^{(6)}_\mathrm{EFT} = {\mathcal {L}}_\mathrm{SM} + \sum _i \frac{\tilde{c}_i}{m_{\scriptscriptstyle W}^2} {\mathcal {O}}_{i} , \end{aligned}$$

where ${\mathcal {L}}_\mathrm{SM}$ stands for the Standard Model Lagrangian. In the above expression, we have normalized the Wilson coefficients $\tilde{c}$ in a way in which the effective scale $\varLambda$ is identified with the *W*-boson mass $m_{\scriptscriptstyle W}$.

The most general ${\mathcal {L}}^{(6)}_\mathrm{EFT}$ Lagrangian invariant under the Standard Model $SU(3)_c \times SU(2)_L \times U(1)_Y$ gauge symmetries has been known for a long time [35–37], and is usually cast in a suitable form by adopting a convenient basis of independent operators [38–41]. In this work, we focus on the dimension-six CPV interactions of the Higgs and the electroweak gauge bosons that are written, in a form inspired by the SILH basis conventions [38, 40], as

2 $$\begin{aligned} {\mathcal {L}}_\mathrm{CP}= & {} i g \frac{\tilde{c}_{\scriptscriptstyle HW}}{m_{\scriptscriptstyle W}^2} D^\mu \varPhi ^\dag T_{2k} D^\nu \varPhi {\widetilde{W}}_{\mu \nu }^k + i g' \frac{\tilde{c}_{\scriptscriptstyle HB}}{m_{\scriptscriptstyle W}^2} D^\mu \varPhi ^\dag D^\nu \varPhi {\widetilde{B}}_{\mu \nu } \nonumber \\&+ g'^2 \frac{\tilde{c}_{\scriptscriptstyle \gamma }}{m_{\scriptscriptstyle W}^2} \varPhi ^\dag \varPhi B_{\mu \nu } {\widetilde{B}}^{\mu \nu } + g_s^2 \frac{\tilde{c}_{\scriptscriptstyle g}}{m_{\scriptscriptstyle W}^2} \varPhi ^\dag \varPhi G_{\mu \nu }^a {\widetilde{G}}^{\mu \nu }_a \nonumber \\&+ g^3 \frac{\tilde{c}_{\scriptscriptstyle 3W}}{m_{\scriptscriptstyle W}^2} \epsilon _{ijk} W_{\mu \nu }^i W^\nu {}^j_\rho {\widetilde{W}}^{\rho \mu k} \nonumber \\&+ g_s^3 \frac{\tilde{c}_{\scriptscriptstyle 3G}}{m_{\scriptscriptstyle W}^2} f_{abc} G_{\mu \nu }^a G^\nu {}^b_\rho {\widetilde{G}}^{\rho \mu c} , \end{aligned}$$

where $B_{\mu \nu }$, $W_{\mu \nu }$ and $G_{\mu \nu }$ ($\widetilde{B}_{\mu \nu }$, $\widetilde{W}_{\mu \nu }$ and $\widetilde{G}_{\mu \nu }$) denote the hypercharge, weak isopsin and strong (dual) field strength tensors, respectively. In addition, $\varPhi$ represents the electroweak doublet of Higgs fields, $g'$, *g* and $g_s$ are the $SU(3)_c$, $SU(2)_L$ and $U(1)_Y$ gauge coupling constants and $\epsilon _{ijk}$ and $f_{abc}$ are the *SU*(2) and *SU*(3) group structure constants. Translations of the ${\mathcal {L}}_\mathrm{CP}$ Lagrangian into any other commonly considered bases [34, 42, 43] can be automatically performed with, e.g., the Rosetta package [44].

**Fig. 1:**
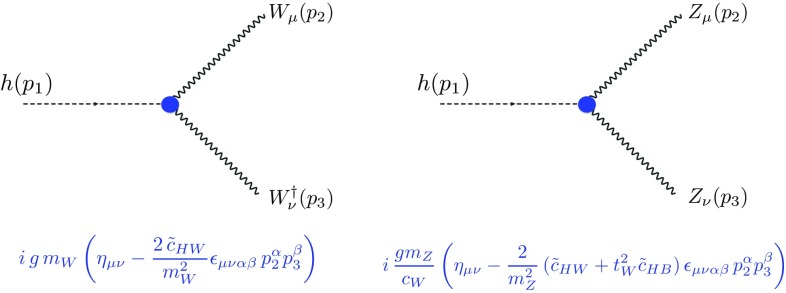
Feynman rules associated with dimension-six CPV operators involving a Higgs boson and a pair of weak bosons

The ${\mathcal {L}}_\mathrm{CP}$ Lagrangian induces new Lorentz structures, such as those featured in the Feynman rules depicted in Fig. 1, which have a manifest CPV structure. Although the restricted set of operators included in Eq. (2) can in principle be extended by CPV fermionic operators [45–49], we postpone the study of the latter to a future work. We moreover consider observables involving a Higgs and/or a weak boson, so that the last operator of Eq. (2) is also irrelevant. The Wilson coefficient parameter space of interest is therefore spanned by the $\{\tilde{c}_g, \ \tilde{c}_\gamma ,\ \tilde{c}_{HW},\ \tilde{c}_{HB},\ \tilde{c}_{3W}\}$ ensemble of free parameters. In principle, the $\tilde{c}_g$ operator could be constrained by multijet processes, like for the corresponding *CP*-even operator. However, this requires a dedicated study, which is beyond the scope of this work.

In general, it is difficult to construct a new physics model that will only induce *CP*-violating operators. On the other hand, the hypothesis of a purely *CP*-odd Higgs boson is experimentally disfavored whereas the experimental bounds on the Higgs boson being an admixture of *CP*-even and *CP*-odd states are very weak [20, 21]. Therefore, a more realistic setup would be a case where the Lagrangian contains both *CP*-odd and *CP*-even operators. Deriving constraints on this new physics configuration would then require a multidimensional fit of all *CP*-odd and *CP*-even parameters. As a first study, we nevertheless consider the purely *CP*-odd Lagrangian of Eq. (2) and leave the joint study of the impact of both *CP*-even and *CP*-odd operators for future work.

The main effects that originate from the $\tilde{c}_{HB}$ operator, however, arise from the Higgs coupling to the *Z*-boson, and can thus always be reabsorbed by a redefinition of the $\tilde{c}_{HW}$ operator,

3 $$\begin{aligned} \tilde{c}_{HW}\rightarrow \tilde{c}_{HW}+ t_{\scriptscriptstyle W}^2 \, \tilde{c}_{HB} , \end{aligned}$$

where $t_{\scriptscriptstyle W}=\tan \theta _{\scriptscriptstyle W}$ is the tangent of the electroweak mixing angle (as shown in the second Feynman rule of Fig. 1).

In order to probe the considered Wilson coefficient parameter space, we study a set of processes that are particularly sensitive to CPV new physics effects in the electroweak sector and that are shown in Table 1, together with their dependence on the different EFT parameters. We consider simulations of collisions such as occurring at the LHC where the hard process is calculated at the leading-order accuracy and the fixed-order result is then matched with parton showers for a proper description of the QCD environment. Detector effects are ignored, as well as next-to-leading order QCD corrections that could in principle imply a dependence on the CPV triple-gluon operator ${\mathcal {O}}_{3G}$.

We can interpret the Lagrangian terms of Eq. (2) as the low-energy manifestation of some new physics arising at a scale $\varLambda$, the details of the ultraviolet completion being encoded in the $\tilde{c}$ coefficients. Denoting by $g_\mathrm{NP}$ the strength of the new physics interactions, one can derive

4 $$\begin{aligned} \frac{\tilde{c}}{m_{\scriptscriptstyle W}^2} \approx \frac{g_\mathrm{NP}^2}{\varLambda ^2} . \end{aligned}$$

This expression approximates the more precise relation that can be computed in an ultraviolet-complete setup, as shown for instance in the analyses of Refs. [50–52]. In the next sections, we adopt the choice of quoting our results in terms of the dimensionless $\tilde{c}$ coefficients, but we also derive a more intuitive estimation of the LHC sensitivity to new physics by extracting a bound on the effective scale $\varLambda$ in the context of typical strongly coupled (so that $\varLambda >\varLambda _s$) and weakly coupled (so that $\varLambda >\varLambda _w$) scenarios. The $\varLambda _s$ and $\varLambda _w$ limits are inferred from Eq. (4), the $g_\mathrm{NP}$ coupling being fixed to $4\pi$ and *g* for the strongly coupled and weakly coupled new physics cases, respectively. Deriving the $\varLambda _w$ and $\varLambda _s$ values enables us to verify whether the phase-space regions probed in our investigations of the CPV operators of Eq. (2) are regions where the EFT approach is reliable. Our test is based on a comparison of the hard scattering scale of the simulated collisions with the $\varLambda _s$ and $\varLambda _w$ values, which differs from other methods that have been proposed to assess the validity of the EFT approach [53, 54]. It should therefore be taken as a matter of convention to translate limits on dimensionless $\tilde{c}$ coefficients to limits on a mass scale. In particular, in theories where new physics effects are only induced at the loop level, additional loop-suppression factors must be incorporated.

**Table 1:** List of LHC processes investigated in this work, presented together with their dependence, indicated by a star, on the EFT operators under consideration

Process	$\tilde{c}_g$	$\tilde{c}_\gamma$	$\tilde{c}_{HW}$	$\tilde{c}_{HB}$	$\tilde{c}_{3W}$
$p p \rightarrow h \rightarrow \gamma \gamma$	$\star$	$\star$			
$p p \rightarrow h \rightarrow Z Z^{(*)} \rightarrow 4 \ell$	$\star$			$\star$	
$p p \rightarrow h \rightarrow Z \gamma$	$\star$		$\star$	$\star$	
$p p \rightarrow Z h \rightarrow \ell ^+ \ell ^- b \bar{b}$			$\star$	$\star$	
$p p \rightarrow Z h \rightarrow \nu \bar{\nu }b \bar{b}$			$\star$	$\star$	
$p p \rightarrow W h \rightarrow \ell \nu b \bar{b}$			$\star$		
$p p \rightarrow h j j$ (VBF)			$\star$	$\star$	
$p p \rightarrow WW \rightarrow \ell \nu \ell ' \nu '$			$\star$		$\star$

## Connecting the effective approach to ultraviolet-complete models

Although the EFT paradigm allows one to pursue a model-independent approach to new physics, it is always important to reinterpret any EFT result in the framework of specific ultraviolet-complete models. Maximizing the chances of discovering new physics motivates to follow pragmatically both a top-down and a bottom–up path. The explicit matching of an ultraviolet-complete theory to its effective counterpart is, however, going beyond the scope of this work.

The simplest example incorporating an ultraviolet origin for the CPV new physics operators of the effective Lagrangian of Eq. (2) consists of a setup where the Standard Model is supplemented by new heavy fermions whose interactions with the Higgs boson feature explicit CPV effects. More precisely, we consider a set of new heavy quarks,

5 $$\begin{aligned} \bigg \{\begin{array}{lll} Q=\begin{pmatrix}T \\ B \end{pmatrix},&T' ,&B'\end{array} \bigg \}, \end{aligned}$$

where *Q* is a weak doublet of hypercharge 1/6, and where $T'$ and $B'$ are two weak singlets of hypercharge 2/3 and − 1/3, respectively. Yukawa interactions of these new fields with the Higgs field $\varPhi$ can be generically written as

6 $$\begin{aligned} {\mathcal {L}}_\mathrm{UV}= & {} - y_B \, \bar{Q} \varPhi B' - i \tilde{y}_B \bar{Q} \varPhi \gamma _5 B' - y_T \, \bar{Q} \cdot \varPhi ^\dag T' \nonumber \\&- i \tilde{y}_T \, \bar{Q} \cdot \varPhi ^\dag \gamma _5 T' + \text {h.c.} , \end{aligned}$$

where the dot product stands for the *SU*(2)-invariant scalar product and where any possible mixing of the Standard Model quarks with the new heavy states is neglected. Such new fermions could appear, for example, in composite Higgs models where fermionic partners to the third generation quarks are introduced to trigger the breaking of the electroweak symmetry [4].

The integration out of the heavy fermions leads to the generation of several effective *CP* violating and *CP* conserving operators. One obtains, for instance, a non-vanishing dimension-six coupling of the Higgs field to the gluon field strength tensor,

7 $$\begin{aligned} {\mathcal {L}}_\mathrm{EFT}= \frac{g_s^2}{16 \pi ^2} \left[ \frac{\tilde{y}_B^2}{m^2_B} + \frac{\tilde{y}_T^2}{m^2_T}\right] \varPhi ^\dag \varPhi \, G_{\mu \nu }^a {\widetilde{G}}^{\mu \nu }_a . \end{aligned}$$

Mapping this operator to the Lagrangian of Eq. (2), one gets the matching condition

8 $$\begin{aligned} \tilde{c}_g = \frac{1}{16 \pi ^2} \left[ \frac{m_{\scriptscriptstyle W}^2}{m^2_B} \tilde{y}_B^2 + \frac{m_{\scriptscriptstyle W}^2}{m^2_T} \tilde{y}_T^2 \right] , \end{aligned}$$

where the new physics coupling strength $g_\mathrm{NP}$ is identified with the CPV Yukawa couplings, and where the new physics scale corresponds to the mass of the heavy fermions.

The operators shown in Eq. (2) can also be generated in compositeness models including composite scalars [55, 56]. Depending on the vacuum structure [57], the *CP* symmetry can be spontaneously broken and yield to CPV EFT operators once the heavy scalars are integrated out [58, 59].

On different grounds, many popular extensions of the Standard Model contain an extended Higgs sector that includes, e.g., new scalar weak singlets or doublets. Explicit CPV in the Higgs sector does not, however, induce effective operators such as those shown in the Lagrangian of Eq. (2), but instead modifies the magnitude of the Standard Model Higgs couplings [51]. Most beyond the Standard Model theories nonetheless generally exhibit a particle spectrum with many new degrees of freedom, whose integration out in contrast leads to new Lorentz structures in the interactions of the Standard Model fields [60–64].

## LHC Run I bounds on CPV EFT operators

Constraints on the Wilson coefficients appearing in the Lagrangian of Eq. (2) can be obtained by analyzing Higgs-boson and vector-boson decay and production rates once predictions in the EFT framework are compared with LHC Run I measurements. The most stringent Run I constraints on the $\tilde{c}_g$ and $\tilde{c}_\gamma$ coefficients arise from the results of the CMS and ATLAS combination for Higgs-boson production and decay in the $g g \rightarrow h \rightarrow \gamma \gamma$ channel [3], the associated signal strength being given by

9 $$\begin{aligned} \mu _\mathrm{LHC}^{gg\rightarrow h\rightarrow \gamma \gamma } = 1.09_{-0.10}^{+0.11} . \end{aligned}$$

While other limits on the new physics contributions to the Higgs-boson couplings to gluons and photons are available, these are extracted under the assumption that either the Higgs-boson width or its production rate is the Standard Model one. We thus restrict ourselves to the use of Eq. (9). The corresponding theoretical predictions (see Appendix A for technical details of the simulations performed in this work) can be fitted by a quadratic function of the CPV $\tilde{c}_g$ and $\tilde{c}_\gamma$ parameters,

10 $$\begin{aligned} \mu _\mathrm{EFT}^{g g \rightarrow h \rightarrow \gamma \gamma }= & {} 1.0 + 2.0 \times 10^{7} \tilde{c}_\gamma ^2 - 1.3 \times 10^3 \tilde{c}_\gamma \tilde{c}_g\nonumber \\&+ 2.0 \times 10^5 \tilde{c}_g^2 , \end{aligned}$$

where the absence of linear terms stems from the vanishing interferences between the new physics and the Standard Model contributions.

On the other hand, electroweak Higgs-boson production processes allow one to constrain both the $\tilde{c}_{HW}$ and the $\tilde{c}_{HB}$ coefficients on the basis of LHC Run I and Tevatron data. Starting with Higgsstrahlung (*VH*) signal strengths, the CVP EFT framework depicted by Eq. (2) leads to theoretical predictions that can be fitted quadratically by

11 $$\begin{aligned} \mu _\mathrm{EFT}^{ZH,\mathrm{~LHC}}= & {} 1.0+145.6 (\tilde{c}_{HW}+t_W^2\tilde{c}_{HB})^2 ,\nonumber \\ \mu _\mathrm{EFT}^{WH,\mathrm{~LHC}}= & {} 1.0+52.3 \tilde{c}_{HW}^2 , \nonumber \\ \mu _\mathrm{EFT}^{ZH,\mathrm{~Tev}}= & {} 1.0+104.7 (\tilde{c}_{HW}+t_W^2 \tilde{c}_{HB})^2 ,\nonumber \\ \mu _\mathrm{EFT}^{WH,\mathrm{~Tev}}= & {} 1.0 + 35.12 \tilde{c}_{HW}^2 , \end{aligned}$$

for the LHC and the Tevatron colliders, respectively. These must be compared with the corresponding measurements [3],

12 $$\begin{aligned} \begin{aligned} \mu _\mathrm{LHC}^{WH}&= 0.88^{+0.40}_{-0.38} , \\ \mu _\mathrm{LHC}^{ZH}&= 0.80^{+0.39}_{-0.36} , \\ \mu _\mathrm{Tev}^{VH}&= 1.59^{+0.69}_{-0.72} , \end{aligned} \end{aligned}$$

the Tevatron value being mainly driven by the *ZH* production mode with a final-state signature containing either zero or two leptons [65, 66]. Additional constraints can be induced by vector-boson fusion (VBF) Higgs production results, and in particular by the *WW* channel (WBF) that contributes to the signal strength result with a weight of 80%. A fit of the EFT theoretical predictions gives

13 $$\begin{aligned} \mu ^\mathrm{WBF,~LHC}_\mathrm{EFT} = 1.0 + 25.3 \, \tilde{c}_{HW}^2 , \end{aligned}$$

which can be confronted with the Run I results,

14 $$\begin{aligned} \mu _\mathrm{LHC}^\mathrm{WBF} = 1.18^{+0.25}_{-0.23} . \end{aligned}$$

Although VBF data is more precise and features smaller error bars than in the *VH* case, the sensitivity of the *VH* production processes to the CPV EFT operators is then expected to be higher than in the VBF case, as pointed out by the numerical factors multiplying the $\tilde{c}$ terms found in Eqs. (11) and (13).

**Table 2:** LHC Run I constraints on the Wilson coefficients associated with the CPV EFT operators given in Eq. (2) (second column), also cast in the form of a bound on the effective scale for strongly coupled (third column) and weakly coupled (fourth column) new physics. The brackets indicate that the limit has been extracted under conditions not compatible with the expected EFT range of validity. We refer to Sect. 2 for details of how the limits on $\varLambda _{w,s}$ are defined

Coefficient	Limit	$\varLambda _s$	$\varLambda _w$
$|\tilde{c}_g|$	1.2 $\times 10^{-4}$	92 TeV	4.4 TeV
$|\tilde{c}_\gamma |$	1.2 $\times 10^{-3}$	29 TeV	1.4 TeV
$|\tilde{c}_{HW}|$	0.06	4.1 TeV	[0.2 TeV]
$|\tilde{c}_{HB}|$	0.23	2.1 TeV	[0.1 TeV]
$|\tilde{c}_{3W}|$	0.18	2.4 TeV	[0.1 TeV]

**Fig. 2:**
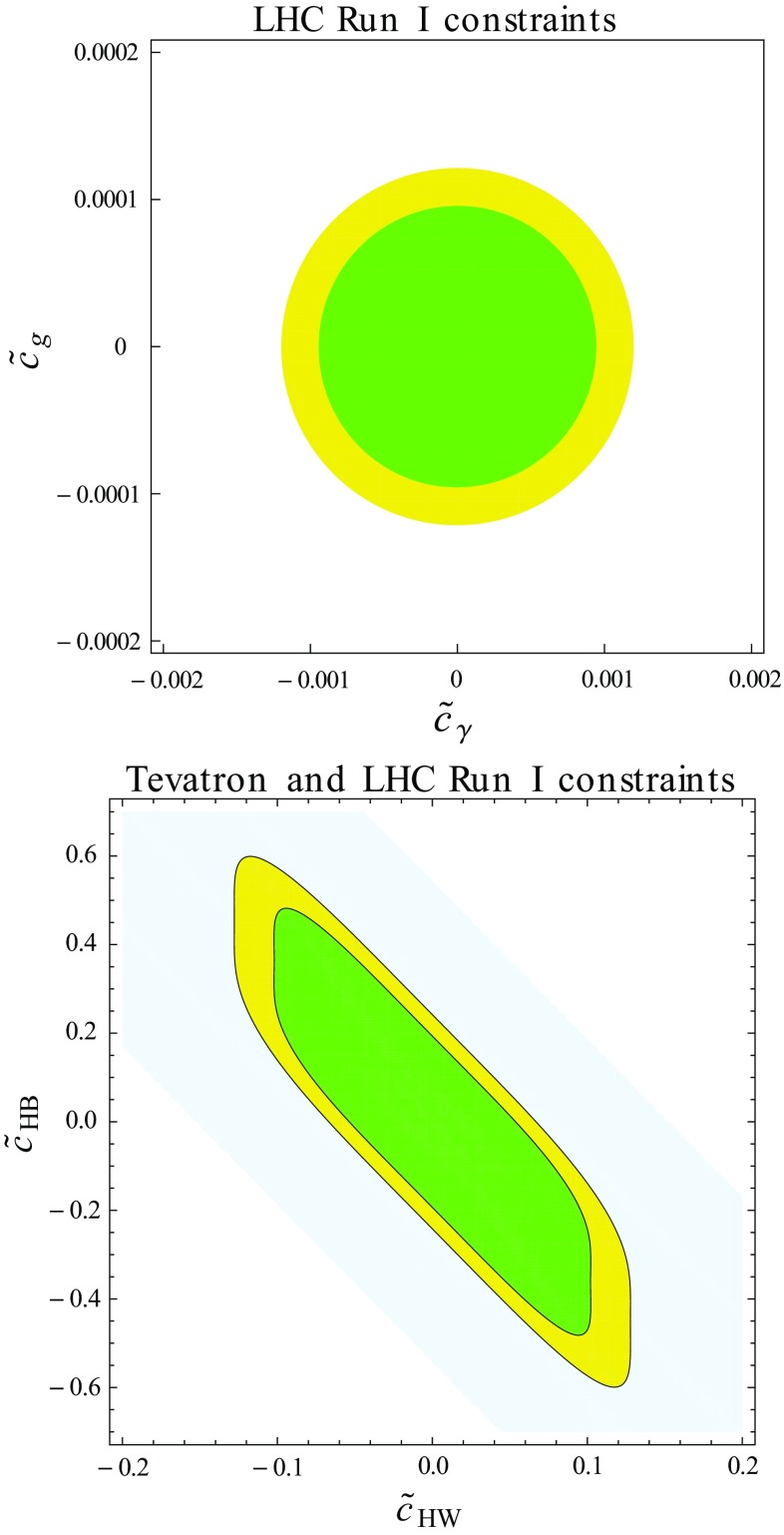
Collider bounds on several of the effective operators considered in the Lagrangian of Eq. (2). We show parameter space regions in agreement with LHC Run I data in the $(\tilde{c}_\gamma , \tilde{c}_g)$ (left) and $(\tilde{c}_{HW}, \tilde{c}_{HB})$ (right) plane at the $1\sigma$ (green) and $2\sigma$ (yellow) level, and the region allowed by Tevatron data at the 95% confidence level is indicated by the blue area

From the relations derived above, we perform a $\chi ^2$ fit of LHC data and extract limits on the effective parameters. The results are shown in Table 2, as well as in Fig. 2 where we have projected them in the $(\tilde{c}_\gamma , \tilde{c}_g)$ (left) and $(\tilde{c}_{HW}, \tilde{c}_{HB})$ (right) planes. Our procedure relies on neglecting the *WH* Tevatron information and on averaging the experimental errors. We observe that operators which affect processes that are loop-suppressed in the Standard Model are more strongly constrained, the maximum allowed value for the associated $\tilde{c}_g$ and $\tilde{c}_\gamma$ parameters being of the order of 0.001 for an effective scale being the *W*-boson mass. Equivalently, this corresponds to probing an effective scale reaching the multi-TeV regime for typical strongly coupled or weakly coupled new physics. In contrast, current limits on the electroweak operators and the corresponding $\tilde{c}_{HW}$, $\tilde{c}_{HB}$ and $\tilde{c}_{3W}$ parameters must be carefully interpreted in the case of weakly coupled new physics. The corresponding bound on the effective scale indeed implies that this scale may be too small to guarantee the validity of the EFT all over the limit extraction procedure. The results finally also depict the strengthening of the Tevatron constraints once LHC Run I measurements are accounted for.

The $\tilde{c}_{HW}$, $\tilde{c}_{HB}$ and $\tilde{c}_{3W}$ are hence currently only loosely constrained by data. In the rest of this work, we demonstrate how future LHC data at a higher center-of-mass energy is expected to provide better handles on the associated operators, and we design novel ways to use the 13 TeV future results to enhance the corresponding LHC sensitivity.

In addition to the processes introduced above, the $\tilde{c}_{HW}$ and $\tilde{c}_{HB}$ parameters could also be constrained by investigating Higgs-boson production and decay into a four-leptonic final state. Fitting the theoretical predictions, the related LHC signal strength is given, in the CPV EFT context, by

15 $$\begin{aligned} \mu _\mathrm{EFT}^{pp\rightarrow h\rightarrow 4\ell , \mathrm{LHC}} = 1.0 + 123.3 (\tilde{c}_{HW}+ t_W^2 \tilde{c}_{HB})^2 , \end{aligned}$$

that we can compare the ATLAS and CMS combined value [3] of

16 $$\begin{aligned} \mu _\mathrm{LHC}^{pp\rightarrow h\rightarrow 4\ell } = 1.13^{+0.34}_{-0.31} . \end{aligned}$$

This process is also strongly affected by the $\tilde{c}_g$ parameter, so that meaningful constraints should be extracted from a multidimensional fit. However, we have verified that the predictions barely depend on this higher-dimensional coupling once its range is restricted by the current constraints. We therefore neglect it in the subsequent analysis.

Table 2 finally also includes a bound on the $\tilde{c}_{3W}$ coefficient that we have extracted from the LHC Run I *W*-boson pair production cross section measurement [67],

17 $$\begin{aligned} \sigma _{WW}= 71.1 \pm 1.1 \ (\mathrm{stat})^{+5.7}_{-5.0} \ (\mathrm{syst}) \pm 1.4 \ (\mathrm{lumi}) \ \mathrm{pb} . \end{aligned}$$

Making use of the Standard Model predictions computed at the next-to-next-to-leading order accuracy in QCD [68–70],

18 $$\begin{aligned} \sigma _{WW}^{(\mathrm{NNLO})} = 63.2^{+1.6}_{-1.4} \ (\mathrm{scale}) \pm 1.2 \ (\mathrm{PDF}) \ \mathrm{pb} , \end{aligned}$$

we can derive a signal strength value $\mu _\mathrm{LHC}^{WW}$ by computing the largest possible allowed deviation in the ratio of data to theory once all errors are added in quadrature [71],

19 $$\begin{aligned} \mu _\mathrm{LHC}^{WW} = 1.13 \pm 0.07 . \end{aligned}$$

This result can then be confronted with the CPV EFT fitted signal strength

20 $$\begin{aligned} \mu _\mathrm{EFT}^{WW} = 1.0 +8.0 \tilde{c}_{3W}^2 . \end{aligned}$$

Additional constraints could also in principle be derived from *WZ* and *ZZ* total cross section measurements, but these are found less sensitive to the considered new physics operators, and are thus ignored.

Experimental collaborations have also performed specific studies on anomalous Higgs couplings to the Standard Model vector bosons in the dilepton and the four-lepton channel [20, 21]. The general line of these analyses relies on Higgs-boson production via gluon fusion, with a subsequent decay of the Higgs boson into a pair of vector bosons. The two channels that have been considered are the $h\rightarrow W^{+}W^{-}\rightarrow 2\ell 2\nu$ and $h\rightarrow ZZ\rightarrow 4\ell$ ones, and the analysis strategy involves several kinematic discriminants being several invariant masses. The results are presented in terms of an effective fractional cross section which describes the allowed amount of deviation with respect to the Standard Model expectation. The Run I results have been found not conclusive due to a too low statistics, and the 13 TeV results still allow for a large amount of *CP*-violation.

## Prospective LHC studies on the basis of inclusive measurements

In this section, we evaluate the LHC sensitivity to new physics effects modeled by the effective operators of the Lagrangian of Eq. (2), assuming an integrated luminosity of either $300~\hbox {fb}^{-1}$ (to be achieved by 2020) or $3000~\hbox {fb}^{-1}$ (the goal of the High-Luminosity LHC program). The estimate of the prospects for the precise determination of the Higgs couplings has been deeply studied by all experimental collaborations, and the ATLAS collaboration has in particular presented results including a channel breakdown [72]. The pieces of information relevant for our study are summarized in Table 3 under the form of the expected precision on the signal strengths corresponding to various Higgs-boson production and decay subprocesses, the theory errors being omitted for brevity.

**Table 3:** Expected accuracy on the Higgs signal strength measurements for different luminosities and different channels, as extracted from Ref. [72]. From a study of the $pp\rightarrow h \rightarrow \gamma \gamma$ process (first block of the table), one can extract constraints on the $\tilde{c}_\gamma$ and $\tilde{c}_g$ parameters. The next four channels (second block of the table) provide information on the $\tilde{c}_\gamma$, $\tilde{c}_g$, $\tilde{c}_{HB}$ and $\tilde{c}_{HW}$ Wilson coefficients while all other processes (last block of the table) probe the $\tilde{c}_{HB}$ and $\tilde{c}_{HW}$ parameters

Channel	$\varDelta \mu /\mu$—$300~\hbox {fb}^{-1}$	$\varDelta \mu /\mu$—3 ab$^{-1}$
$h \rightarrow \gamma \gamma$ (jet veto)	0.13 (0.09)	0.09 (0.04)
$h \rightarrow ZZ$ (gluon fusion)	0.12 (0.07)	0.11 (0.04)
$h \rightarrow WW$ (jet veto)	0.18 (0.09)	0.16 (0.05)
$h \rightarrow \gamma \gamma$ (VBF)	0.47 (0.43)	0.22 (0.15)
$h \rightarrow \gamma \gamma$ (*WH*)	0.48 (0.48)	0.19 (0.17)
$h \rightarrow Z Z$ (*VH*)	0.35 (0.34)	0.13 (0.12)
$h \rightarrow Z Z$ (VBF)	0.36 (0.33)	0.21 (0.16)
$h \rightarrow WW$ (VBF)	0.21 (0.20)	0.15 (0.09)
$h \rightarrow b \bar{b}$ (*ZH*)	0.29 (0.29)	0.14 (0.13)
$h \rightarrow b \bar{b}$ (*WH*)	0.57 (0.56)	0.37 (0.36)

The information embedded in the table allows for a global fit of all the Wilson coefficients included in the Lagrangian of Eq. (2). The three sets of processes under consideration (separated by horizontal lines in the table) can, however, be used to set bounds on independent pairs of operators, which motivates the simpler procedure adopted in the following. For instance, a precise measurement of the Higgs-boson properties in the $pp\rightarrow h\rightarrow \gamma \gamma$ channel, which is dominated by gluon-fusion production, would provide information on the pair of $\tilde{c}_\gamma$ and $\tilde{c}_g$ parameters whereas investigations of VBF or *VH* Higgs-boson production events where the Higgs boson decays into a weak-boson pair or a $b\bar{b}$ pair yield independent information on the $\tilde{c}_{HB}$ and $\tilde{c}_{HW}$ parameters. As a consequence, we focus on two-dimensional fits that are also easier to represent.

**Fig. 3:**
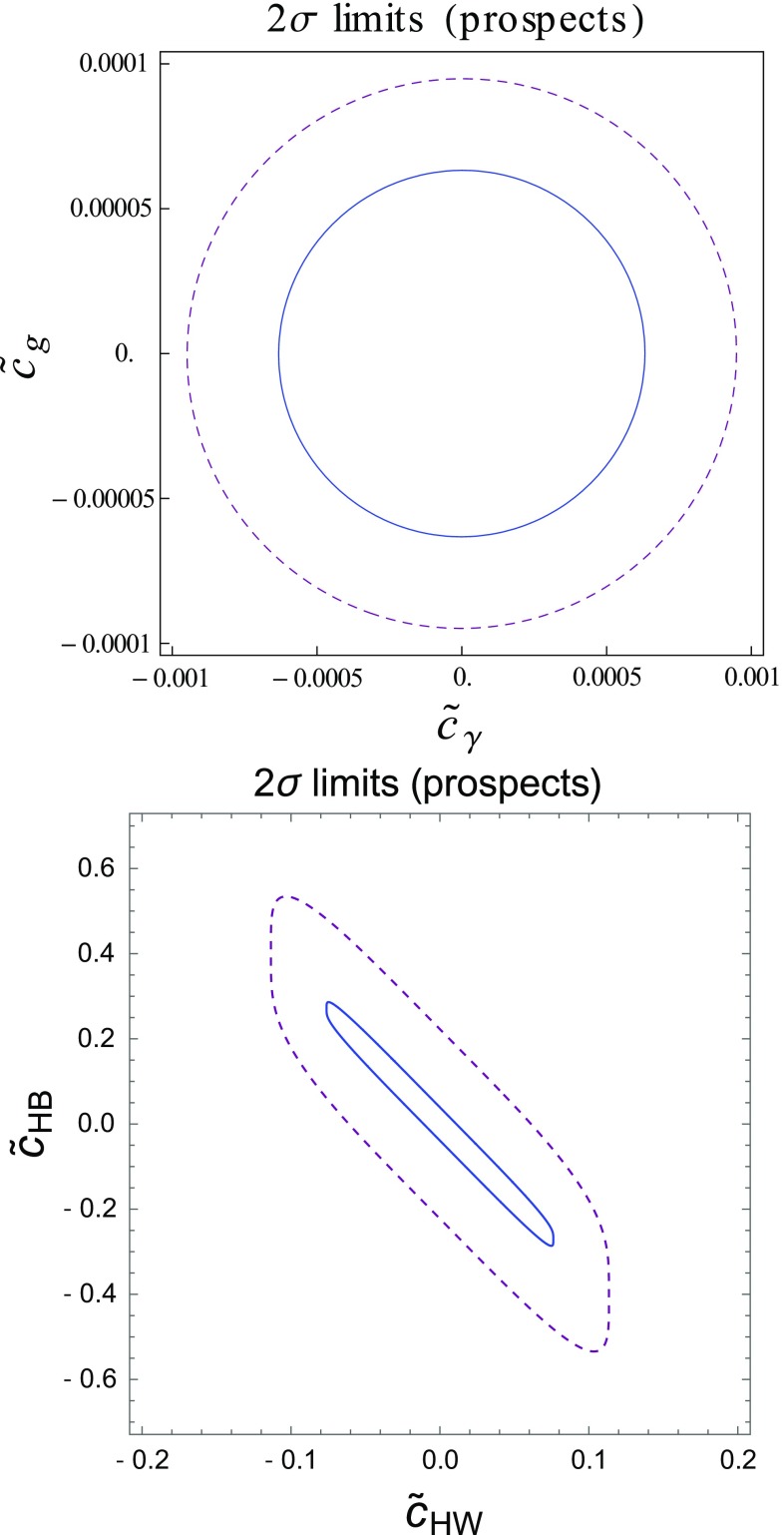
LHC sensitivity to the $\tilde{c}_\gamma$ and $\tilde{c}_g$ (left) and on the $\tilde{c}_{HW}$ and $\tilde{c}_{HB}$ parameters (right). We show the 95% confidence level reach for an integrated luminosity of $300~\hbox {fb}^{-1}$ (dashed purple) and $3000~\hbox {fb}^{-1}$ (solid blue), neglecting the effects of the theoretical uncertainties

Theoretical predictions for the signal strength associated with the $g g\rightarrow h\rightarrow \gamma \gamma$ channel are given, in terms of the $\tilde{c}_g$ and $\tilde{c}_\gamma$ parameters, by the quadratic fitting function

21 $$\begin{aligned} \mu _\mathrm{EFT}^{gg\rightarrow h\rightarrow \gamma \gamma }= & {} 1.0 + 2.0 \times 10^5 \tilde{c}_{\gamma }^2 - 1.5 \times 10^4 \tilde{c}_{\gamma } \tilde{c}_{g} \nonumber \\&+ 2.0 \times 10^7 \tilde{c}_{g}^2 , \end{aligned}$$

once a basic selection is applied on the signal. Confronting those predictions with the expectations presented in Table 3 thus allows one to extract the LHC sensitivity to the $\tilde{c}_g$ and $\tilde{c}_\gamma$ Wilson coefficients. We show results in the left panel of Fig. 3 for a luminosity of 300 fb$^{-1}$ (dashed purple) and $3000~\hbox {fb}^{-1}$ (solid blue) of proton–proton collisions at a center-of-mass energy of 13 TeV.

Similarly, we can extract bounds on the remaining coefficients by focusing on processes independent of the $\tilde{c}_\gamma$ and $\tilde{c}_g$ parameters like those presented in the last panel of Table 3. The predictions for the three most relevant signal strengths are given by

22 $$\begin{aligned} \mu _\mathrm{EFT}^{pp\rightarrow ZH}= & {} 1.0 + 168 (\tilde{c}_{HW}+ t_W^2 \tilde{c}_{HB})^2 , \nonumber \\ \mu _\mathrm{EFT}^{pp\rightarrow WH}= & {} 1.0 + 53 \tilde{c}_{HW}^2 , \nonumber \\ \mu _\mathrm{EFT}^\mathrm{WBF}= & {} 1.0+ 38 \tilde{c}_{HW}^2 . \end{aligned}$$

Besides the channels described above, measurements related to the rare $h\rightarrow Z \gamma$ decay also allow for the extraction of constraints on the $\tilde{c}_{HW}$ and $\tilde{c}_{HB}$ parameters, as the corresponding signal strength is sensitive to these two EFT operator coefficients,

23 $$\begin{aligned} \mu _\mathrm{EFT}^{h\rightarrow Z\gamma } = 1+ 6100 \, (\tilde{c}_{HW}+t_w^2 \tilde{c}_{HB})^2 . \end{aligned}$$

The prospects on limit setting by studying this rare Higgs-boson decay mode have been evaluated for $3000~\hbox {fb}^{-1}$ of LHC collisions [73],

24 $$\begin{aligned} \mu _\mathrm{LHC}^{h\rightarrow Z\gamma } = 1.00^{+0.25}_{-0.26}~\mathrm{(stat.)}~{}^{+0.17}_{-0.15}~\mathrm{(syst.)} , \end{aligned}$$

so that the predictions can be compared to the experimental expected value.

The resulting constraints on the $\tilde{c}_{HB}$ and $\tilde{c}_{HW}$ parameters are shown on the right panel of Fig. 3, when all the channels described above are accounted for.

On different grounds, the $\tilde{c}_{3W}$ coefficient can be constrained as indicated in Sect. 4, on the basis of *W*-boson pair production total rates. Predictions for the corresponding signal strength read

25 $$\begin{aligned} \mu _\mathrm{EFT}^{WW} = 1.0 + 9.3 \tilde{c}_{3W}^2 . \end{aligned}$$

The precision on the related experimental expectation is however tightly bound both to experimental effects and to the accuracy of the theoretical predictions that is currently the next-to-next-to- leading order in QCD [74]. We can optimistically estimate the total error to be of the order of 5%, which would lead to a moderate enhancement of the expected constraints on $\tilde{c}_{3W}$ by a factor of about 2 with respect to the results of Table 2.

Comparing the Run I results (Fig. 2) with the high-luminosity LHC prospects (Fig. 3), we observe that an improvement of a factor of about 2 can be expected. While this mild strengthening of the constraints implies that the EFT is still used in a range where it is valid, this also shows that the current bounds will not drastically change during the next 20 years when solely signal strengths are used. In the next section, we will show how a more dramatic improvement could be achieved by making use of differential distributions. For specific channels like the *VH* or the diboson ones, differential information is actually expected to be more powerful than what could be obtained from total rate measurements [10–12, 75].

## Prospective LHC studies using differential information

Derivative EFT operators have a momentum dependence, illustrated in the Feynman rules of Fig. 1, that could be exploited by focusing on phase-space regions where the momentum transfer is large. As the $\tilde{c}_g$ and $\tilde{c}_\gamma$ Wilson coefficients are already well cornered by total rate measurements in the Higgs-boson dominant production (gluon-fusion) mode once a decay into photons is accounted for, we move on with the use of differential distributions to design an analysis allowing one to improve the expectation on the $\tilde{c}_{HW}$, $\tilde{c}_{HB}$ and $\tilde{c}_{3W}$ parameters. These are all currently relatively less constrained by total rates, and the future prospects have not been found very exciting.

A complication may arise from the fact that in general, as stated in Sect. 2, the EFT Lagrangian stemming from an ultraviolet-complete theory contains both *CP*-even and *CP*-odd operators. One must thus in principle construct observables that genuinely capture the CPV effects. Some extensive studies along these lines have been conducted in previous works [76, 77], where key observables are designed on the basis of triple products of momenta. This has been shown to be sensitive to the interactions of the Higgs boson with a pair of weak gauge bosons. On different lines, the EFT derived from many ultraviolet-complete models, like supersymmetry or the two-Higgs-doublet model, features effective couplings of the Higgs boson to a gauge-boson pair whose CPV component is loop-suppressed. As a consequence, the CPV contributions to cross sections, which are also the quantities usually constrained by previous experimental searches, are always small. Exceptions exist for cases where there is a large admixture of *CP*-odd and *CP*-even states that can be degenerate, and/or when the theory exhibits large *CP*-violating phases [78, 79]. Earlier studies have also attempted to construct angular variables that directly probe the interferences between the *CP*-odd, *CP*-even and the Standard Model contributions in the VBF production mode [80] as well as those induced by the coupling of the Higgs boson to a pair of *Z*-bosons [81].

Another option to get sensitivity to *CP*-violation effects may rely on the usage of phase-space correlations, which may become feasible as more data is being recorded by the experiments. A variety of decay modes could be considered [15]. For instance, the diphoton $h\rightarrow \gamma \gamma$ channel could be promising provided that the photon polarization, a quantity directly related to *CP* violation, could be measured. This can be achieved through the study of the opening azimuthal angle between the two photons, which is expected to be in the [$10^{-4}$–$10^{-3}$] range and that thus lies at the resolution limit of the ATLAS and CMS pixel detectors. It is thus possible to observe substantial effects in parts of the phase space by choosing suitable cuts, but this is unrealistic at the moment as the LHC integrated luminosity is still limited. Another example concerns *Wh* production, but this requires to be able to separate the different initial-state helicity combinations. This can be performed through severe selections necessary as the $q\bar{q}$ initial state is symmetric in the context of a *pp* collision.

**Fig. 4:**
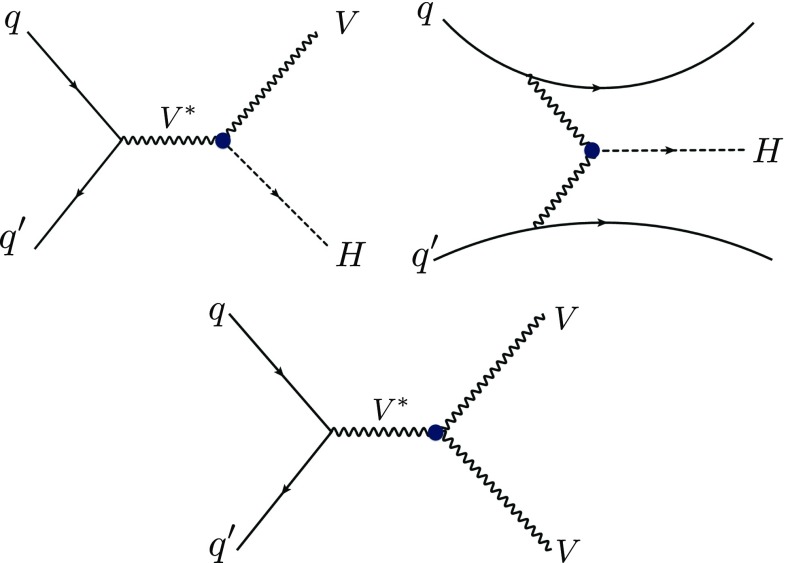
Representative Feynman diagrams for the considered Higgs and weak-boson production mechanisms, namely for *VH* associated production (left), VBF Higgs-boson production (center) and diboson production (right)

To study these momentum-dependent couplings in LHC collisions at a center-of-mass energy of 13 TeV, we consider the electroweak processes shown in Fig. 4, where Higgs and/or weak bosons are produced possibly in association with jets. More precisely, we investigate the associated production of a Higgs and a weak boson (*VH*), Higgs-boson production by vector-boson fusion and diboson production (*VV*). Concerning the boson decays, we consider both the four-lepton mode traditionally studied for *CP*-violation analyses [30–33] and novel channels, the seeds for some of them having been introduced in earlier works [82–89].

Technical details of the LHC collision simulations that we have performed are given in Appendix A.

### *VH* Higgs and weak-boson associated production

In the following, we focus on the associated production of a Higgs and a weak boson when the weak boson decays into either a single-lepton or a dilepton final state. The Higgs boson is additionally considered to decay into a final-state system from which it could be fully reconstructed, the precise definition of this system being therefore not relevant.

**Fig. 5:**
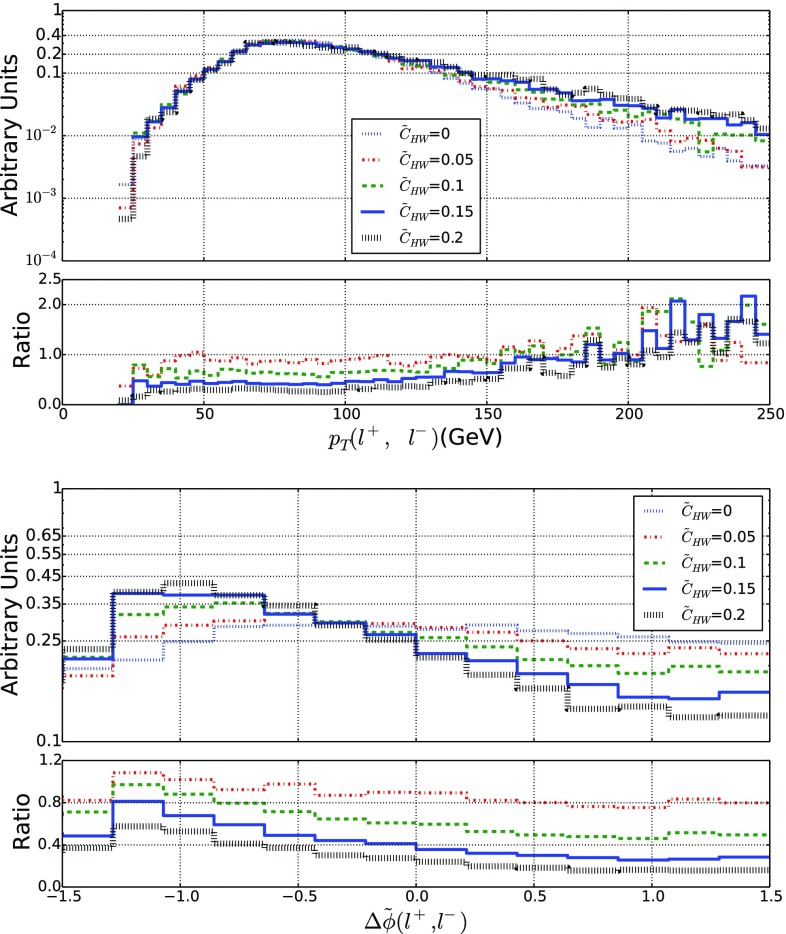
Representative kinematical properties of a dilepton system issued from the decay of a *Z*-boson when the latter is produced in association with a Higgs boson in LHC collisions at a center-of-mass energy of 13 TeV. We consider the scalar sum of the transverse momenta of the two leptons (top) and their angular separation in azimuth (bottom). We allow for different values for the $\tilde{c}_{HW}$ parameter and we present, in the lower panels, the bin-by-bin ratio of the new physics predictions to the Standard Model expectation

When the Higgs boson is produced together with a leptonic *Z*-boson, we can make use of the kinematical properties of the two final-state leptons to get handles on any possible EFT deviation. This is illustrated by the two distributions shown in Fig. 5, namely the scalar sum of the transverse momenta of the two leptons $\ell ^+$ and $\ell ^-$ (upper panel),

26 $$\begin{aligned} p_T(\ell ^+, \ell ^-) = p_T({\ell ^+}) + p_T({\ell ^-}) , \end{aligned}$$

and their angular separation in azimuth (lower panel) defined by

27 $$\begin{aligned} \varDelta \tilde{\phi }(\ell ^+,\ell ^-) = |\varDelta \phi (\ell ^+,\ell ^-)|-\frac{\pi }{2} . \end{aligned}$$

In the Standard Model, the $p_T(\ell ^+, \ell ^-)$ distribution exhibits first a peak for $p_T(\ell ^+, \ell ^-)\sim 60$ GeV before it slowly falls down for larger values. We then allow for a positive non-vanishing $\tilde{c}_{HW}$ parameter varying in the range [0, 0.2]. Although this extends the range allowed by the current constraints when EFT operators are considered one-by-one (see Table 2), this conservatively accounts for potentially weaker constraints that could stem from a EFT fit. We observe that the EFT effects tame the decrease of the distribution for large $p_T(\ell ^+, \ell ^-)$ values, as a result of the enhanced EFT impact when the momentum transfer is large. Deviations of a factor of up to two are found, while one still lies within the EFT range of validity. Other EFT operators could also affect the predictions, like the ${\mathcal {O}}_{HW}$ and ${\mathcal {O}}_{HB}$ operators of the Lagrangian of Eq. (2), and the obtained behavior turns out to be similar. This suggests one to define, as a handle for characterizing new physics, the efficiency $\varepsilon (\tilde{c}, p_T^\mathrm{cut})$ that depends on the Wilson coefficient $\tilde{c}$ and on a minimum value $p_T^\mathrm{cut}$ for the $p_T(\ell ^+, \ell ^-)$ observable,

28 $$\begin{aligned} \varepsilon ({\tilde{c}}) = \frac{1}{\sigma (\tilde{c})} \int _{p_T^\mathrm{cut}}^\infty \frac{\mathrm{d} \sigma (\tilde{c})}{\mathrm{d} p_T(\ell ^+, \ell ^-)} \ \mathrm{d} p_T(\ell ^+, \ell ^-) . \end{aligned}$$

As our simulation is performed at the leading-order accuracy, uncertainties are expected to be large. Although the $\varepsilon$ quantity exhibits a ratio, the cancellation of the uncertainties is only partial as the phase-space cuts are different for the numerator and the denominator. More accurate estimates require the computation of higher-order corrections as well as the resummation of the Sudakov logarithms that are potentially significant for large $p_T$ values.

On the lower panel of Fig. 5, we investigate the angular separation of the two leptons and observe that the EFT effects distort the shape of the spectrum that is more uniform in the Standard Model than when EFT effects are included. A shape analysis going beyond the scope of this paper, we instead define the asymmetry

29 $$\begin{aligned}&{\mathcal {A}}_{\varDelta \tilde{\phi }}(\tilde{c}) \nonumber \\&\quad = \frac{ \mathrm{d}\sigma (\varDelta \tilde{\phi }(\ell ^{+}, \ell ^{-})<0 ) - \mathrm{d}\sigma (\varDelta \tilde{\phi }(\ell ^{+}, \ell ^{-})>0 )}{ \mathrm{d}\sigma (\varDelta \tilde{\phi }(\ell ^{+}, \ell ^{-})<0 ) + \mathrm{d}\sigma (\varDelta \tilde{\phi }(\ell ^{+}, \ell ^{-})>0 )} , \end{aligned}$$

which we use as a second handle on CPV new physics effects, in addition to the $\varepsilon$ variable defined by Eq. (28). In the right-hand side of the above expression, the dependence on the Wilson coefficient is understood for clarity.

**Fig. 6:**
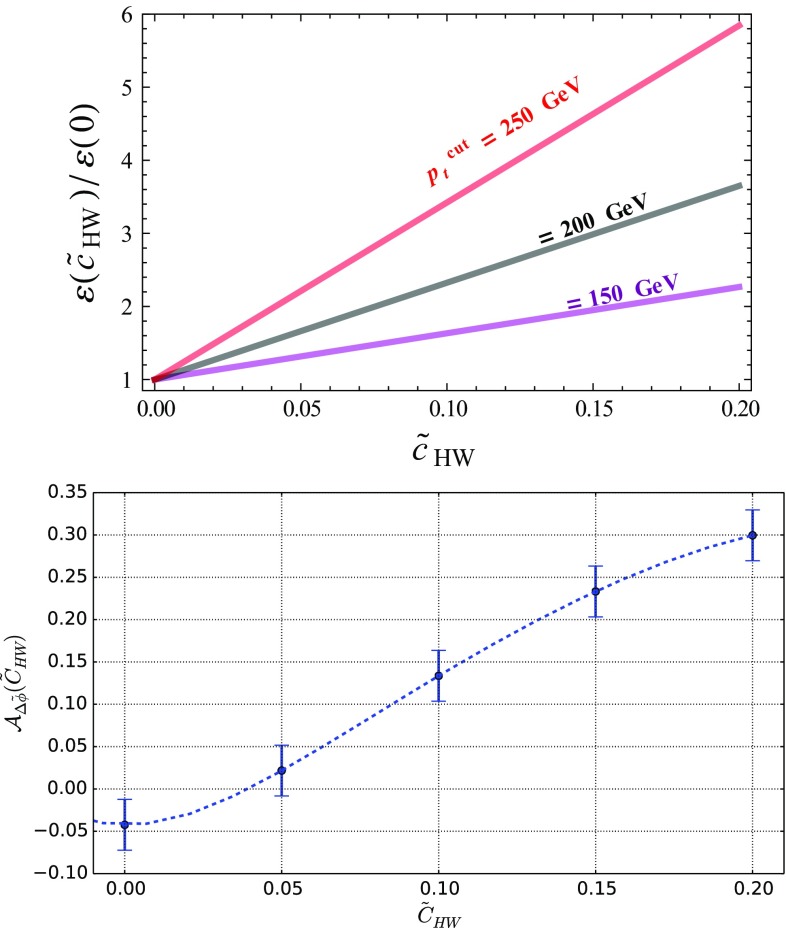
$\tilde{c}_{HW}$ dependence of the $\varepsilon$ variable defined in Eq. (28) (left) for different choices of the $p_T^\mathrm{cut}$ threshold, and of the asymmetry defined in Eq. (29) (right)

**Fig. 7:**
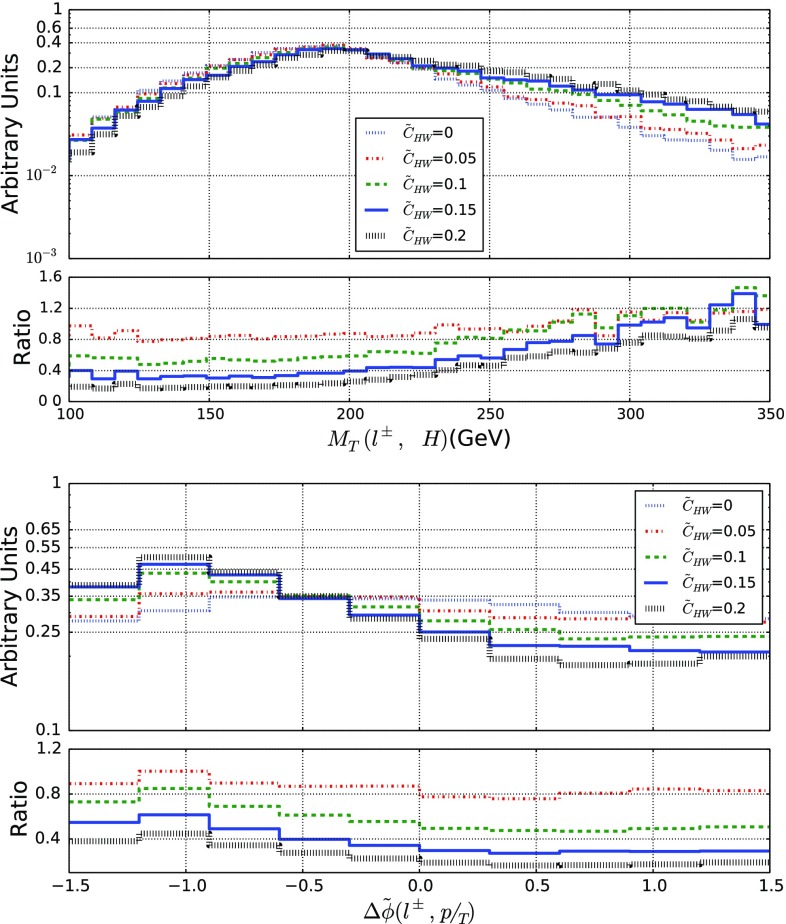
Representative kinematical properties of the decay product of a *WH* system produced in LHC collisions at a center-of-mass energy of 13 TeV. We consider the transverse mass of the *WH* system (top) and the angular separation in azimuth between the lepton and the missing momentum (bottom). We allow for different values for the $\tilde{c}_{HW}$ parameter and we present, in the lower panels, the bin-by-bin ratio of the new physics predictions to the Standard Model expectation

The dependence of the $\varepsilon$ and ${\mathcal {A}}_{\varDelta \tilde{\phi }}$ observables on the $\tilde{c}_{HW}$ parameters is presented in Fig. 6. As expected, a harder selection on $p_T(\ell ^+, \ell ^-)$ implies a larger sensitivity to the EFT operators through the $\varepsilon$ variable, so that it offers a way to probe smaller values of the $\tilde{c}_{HW}$ parameter. Conclusive statements should, however, also account for the reduction of the fiducial cross section, and hence depend on the considered luminosity and the appropriately designed event selection strategy. The ${\mathcal {A}}_{\varDelta \tilde{\phi }}$ asymmetry moreover, shows that large deviations from the Standard Model could be expected, including a possible different sign for some $\tilde{c}_{HW}$ values. Measuring such an observable with a reasonable precision could therefore yield an extra way to constrain EFT deviations.

The Higgs boson could also be produced in association with a *W*-boson, which leads to a final state containing a single lepton once a *W*-boson leptonic decay is accounted for. We again construct appropriate observables that allow for the extraction of bounds on the EFT parameters. In Fig. 7, we show, in the upper panel, the distribution in the transverse mass of the lepton and the reconstructed Higgs-boson system, $M_T(\ell , H)$, and the angular separation in azimuth between the lepton and the missing transverse momentum  (lower panel), this last observable being defined similarly to Eq. (27).

**Fig. 8:**
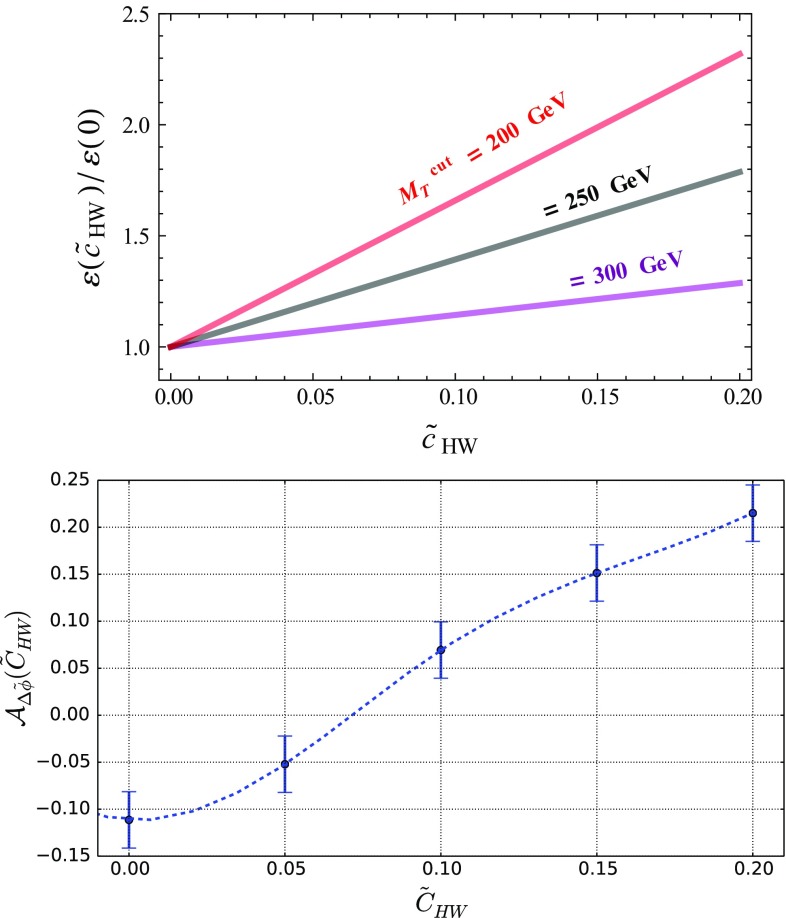
Same as in Fig. 6 but for *WH* production

We observe effects that are similar to the *ZH* case, the EFT operators under consideration impacting the tail of the invariant-mass distribution whose fall at large $M_T(\ell , H)$ values is tamed and yielding a more pronounced shape for the  spectrum. We define an $\varepsilon$ efficiency analogously to Eq. (28),

30 $$\begin{aligned} \varepsilon ({\tilde{c}}) = \frac{1}{\sigma (\tilde{c})} \int _{M_T^\mathrm{cut}}^\infty \frac{\mathrm{d} \sigma (\tilde{c})}{\mathrm{d} M_T(\ell , H)} \ \mathrm{d} M_T(\ell , H) , \end{aligned}$$

which now depends on the Wilson coefficients and on the $M_T^\mathrm{cut}$ minimum value for the transverse mass, as well as an asymmetry as in Eq. (29),31We obtain the results represented in Fig. 8, from which we observe that all *VH* modes offer extra means to constrain CPV operators, the *WH* channel, however, benefiting from a larger cross section so that it could be in principle more promising.

**Fig. 9:**
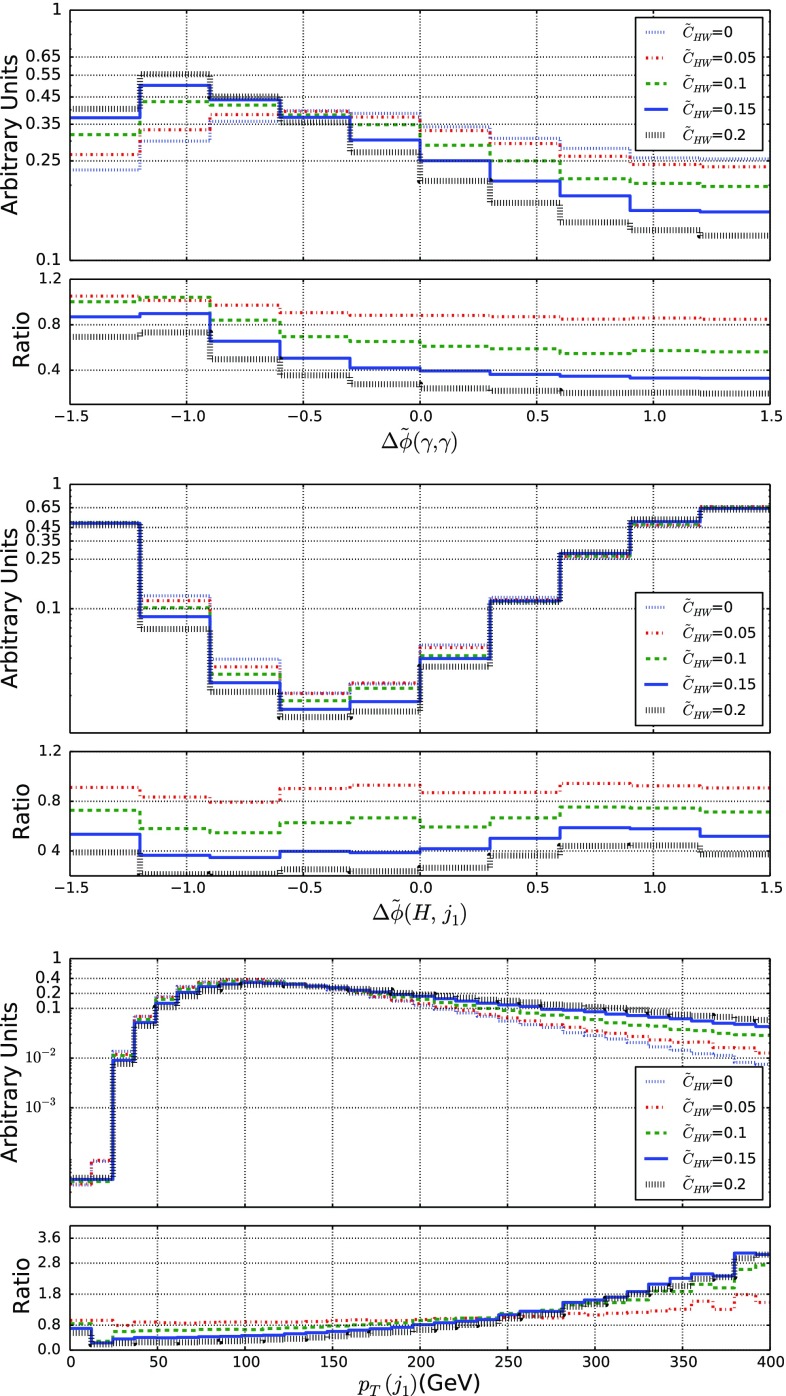
Representative kinematical properties of the decay product of a Higgs boson produced by vector-boson fusion and that decays into a photon pair when produced in LHC collisions at a center-of-mass energy of 13 TeV. We consider the angular separation in azimuth between the photon pair originating from the Higgs-boson decay (top), the angular separation in azimuth between the reconstructed Higgs boson and the leading jet (center) and the transverse momentum of the leading jet (bottom). We allow for different values for the $\tilde{c}_{HW}$ parameter and we present, in the lower panels, the bin-by-bin ratio of the new physics predictions to the Standard Model expectation

### Higgs production by vector-boson fusion

Vector-boson Higgs-boson production processes are excellent probes of physics beyond the Standard Model, in particular when new physics is parameterized within the EFT framework. We focus on three variables which we have found very sensitive to CPV EFT operators, namely the angular separation in the transverse plane $\varDelta \tilde{\phi }(\gamma , \gamma )$, between the decay products of the Higgs boson (considered to be a photon pair), the transverse momentum of the leading forward jet $p_T(j_1)$ and the angular separation in the transverse plane $\varDelta \tilde{\phi }(H, j_1)$ between the reconstructed Higgs boson and the leading forward jet. The distributions in these three observables are shown in Fig. 9, where we observe a standard EFT behavior. The transverse-momentum spectrum of the leading forward jet departs from the Standard Model expectation for large $p_T$ values, the distribution being then harder, and the shapes of the two angular variable distributions is distorted, the effects being more pronounced for $\varDelta \tilde{\phi }(\gamma , \gamma )$. We have verified that these effects are also observed in observables for which we have not presented the results, like the distribution in the transverse momentum of the Higgs boson $p_T (H)$, which is actually strongly correlated to the one of the leading forward jet. The enhancement in the tail of the spectrum is, moreover, also correlated with the suppression of events featuring a large angular separation. Additional information can be obtained by studying the $\varDelta \tilde{\phi }(H, j_1)$ spectrum for $\varDelta \tilde{\phi }$ values in the $[- \ 1.25, 0.25]$ range.

**Fig. 10:**
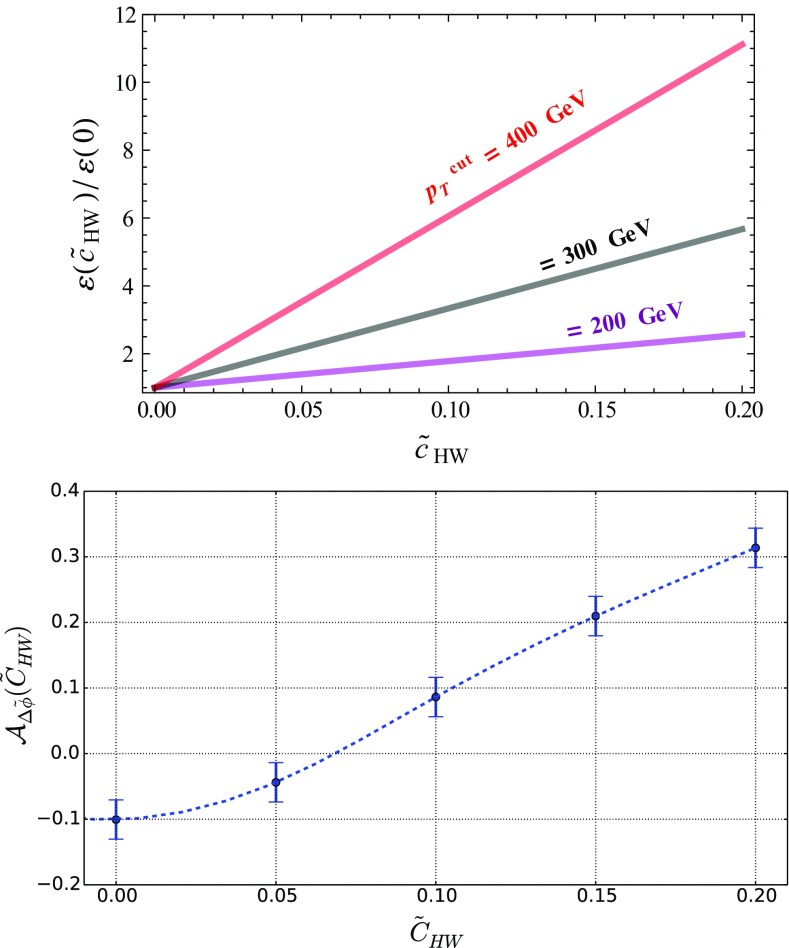
Same as in Fig. 6 but for VBF Higgs-boson production and the observables considered in Sect. 6.2

We define asymmetries (for the angular variables) and efficiencies (for the dimensionful variable) as in the previous section so that these observable can be used for extracting constraints on EFT operators. This is confirmed by the results presented in Fig. 10. We have in particular found a stronger dependence of the asymmetry connected to the Higgs-boson decay products.

### CPV EFT effects in dileptonic *W*-boson pair production events

While all previously considered processes allow us to get information on the ${\mathcal {O}}_g$, ${\mathcal {O}}_\gamma$, ${\mathcal {O}}_{HW}$ and ${\mathcal {O}}_{HB}$ operators, the ${\mathcal {O}}_{3W}$ operator can instead only be constrained by the study of *W*-boson pair production, as already shown in Sects. 4 and 5. We focus on a final-state signature made of two leptons and missing energy, each *W*-boson hence decaying leptonically. After examining several distributions, we have found that the EFT effects are particularly important in the distribution in the invariant mass of the dilepton system $M(\ell ^+ \ell ^-)$, as well as in an analogous of the $\mathcal {O}_{1}$ observable introduced in the context of four-leptonic decays of the Higgs boson [76, 77],

32 $$\begin{aligned} \tilde{\mathcal {O}}_{1} = \frac{\mathbf {p}_{+}\times \mathbf {p}_{-}}{\vert \mathbf {p}_{+}\times \mathbf {p}_{-}\vert } \mathrm{sign} [(\mathbf {p}_{+}-\mathbf {p}_{-}) \cdot \hat{\mathbf z} ] , \end{aligned}$$

where $\mathbf {p}_{\pm }$ denotes the three-momentum of the lepton $\ell ^\pm$ and $\hat{\mathbf z}$ is a unit vector along the collision axis.

**Fig. 11:**
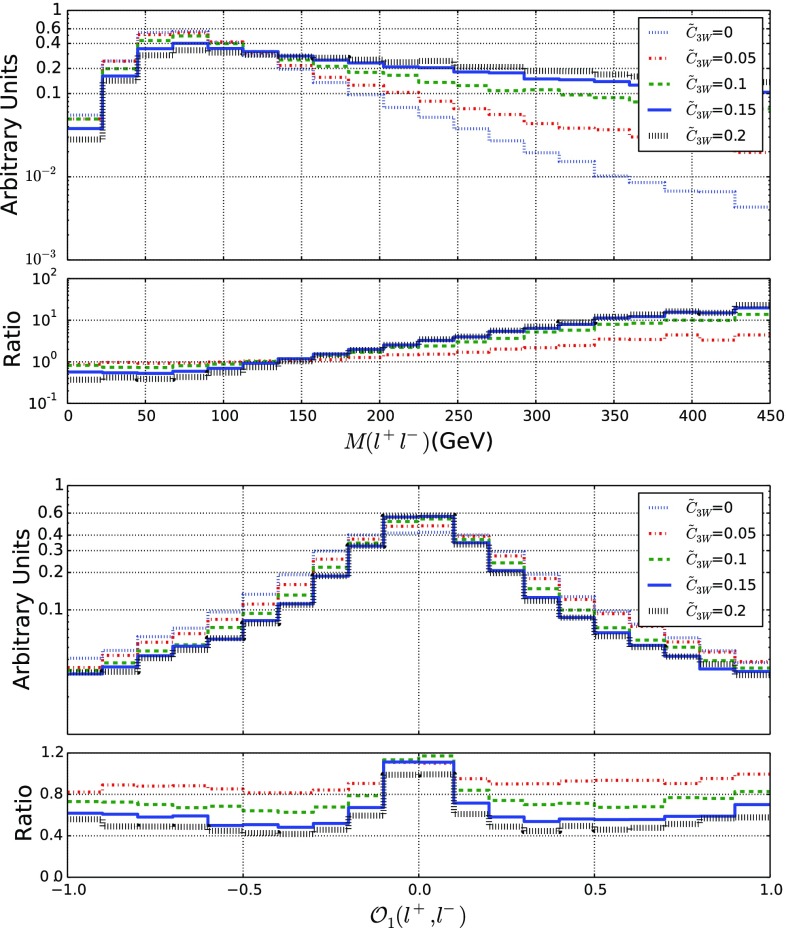
Representative kinematical properties of the decay products of a *W*-boson pair produced in LHC collisions at a center-of-mass energy of 13 TeV. We consider the invariant mass of the dilepton pair issued from the *WW* system (top) and the $\tilde{\mathcal {O}}_1$ observable defined by Eq. (32) (bottom). We allow for different values for the $\tilde{c}_{3W}$ parameter and we present, in the lower panels, the bin-by-bin ratio of the new physics predictions to the Standard Model expectation

We present predictions for the two selected observables in Fig. 11 for different values of the $\tilde{c}_{3W}$ Wilson coefficient. Once again, the tail of the spectrum in the dimensionful $M(\ell ^+ \ell ^-)$ variable turns out to be very sensitive of EFT effects, the distribution becoming harder, and the shape of the spectrum in the $\tilde{\mathcal {O}}_{1}$ observable is modified with respect to the Standard Model case. Similarly to the previous section, we could encapsulate these differences in the definition of an efficiency and an asymmetry that would provide handles on the effective parameters.

### Revisiting CPV Higgs-boson studies in the four-lepton final state

Traditionally, studies of *CP* violation in the Higgs sector have been mostly focused on the four-lepton final state originating from a Higgs-boson decay into a *Z*-boson system [30–33, 89]. In this section, we revisit those studies and show how including appropriate selections could enhance the sensitivity to the EFT operators of the Lagrangian of Eq. (2). We start our analysis by performing an event selection that requires the presence of two pairs of leptons with an opposite electric charge. The invariant mass of the first lepton pair denoted by $Z_1$ is imposed to lie in the [75, 105] GeV range, whilst the one of the second lepton pair denoted by $Z_2$ is enforced to be included in the [10, 200] GeV mass window. The first lepton pair is hence identified with an on-shell *Z*-boson, and the second pair corresponds to the off-shell *Z*-boson issued from the Higgs-boson decay.

**Fig. 12:**
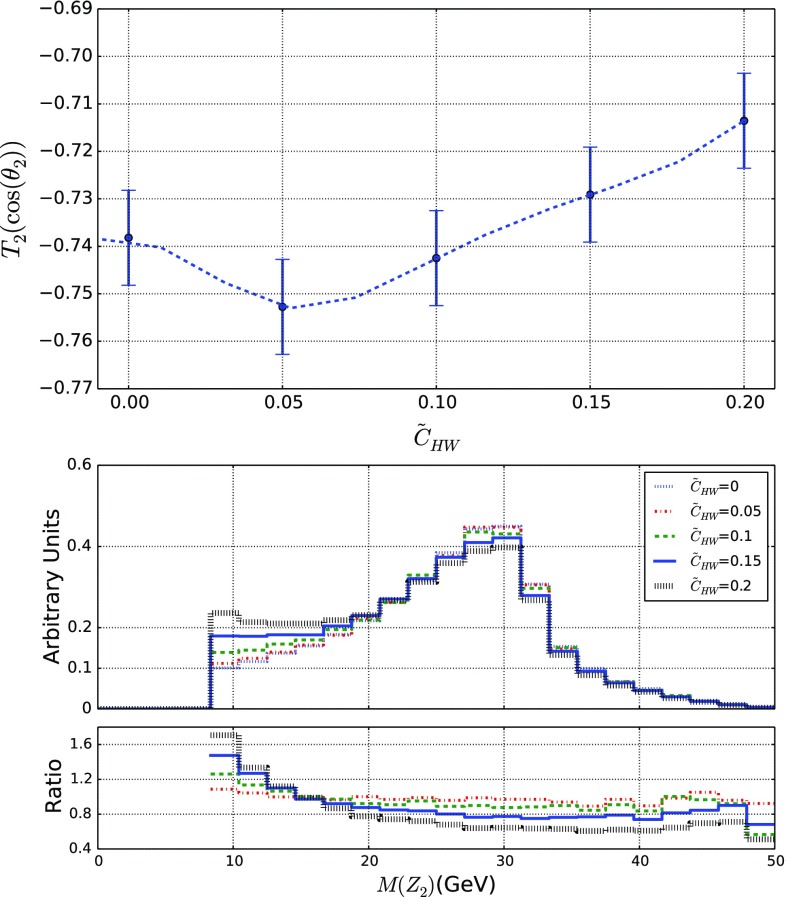
Representative kinematical properties of the four-lepton system originating from a Higgs boson that is decaying into a *Z*-boson pair and that has been produced in LHC collisions at a center-of-mass energy of 13 TeV. We consider the $T_2(\cos \theta _2)$ variable as defined in the text and present its dependence on the $\tilde{c}_{HW}$ parameter, together with the one of the off-shell *Z*-boson invariant-mass distribution (bottom), for varied $\tilde{c}_{HW}$ values. In this last case, we also show, in the lower inset of the figure, the bin-by-bin ratio of the new physics predictions to the Standard Model expectation

Key observables for CPV studies include the polar angles of the leptons, $\theta _{1}$ and $\theta _{2}$, evaluated in the rest frame of the parent $Z_1$ and $Z_2$ bosons, as well as the azimuthal angle $\varphi$ between the two planes formed by the lepton pairs in the Higgs-boson rest frame. Exploring the traditional variables, we have observed that a particular function of the lepton polar angles,

33 $$\begin{aligned} T_2(x)= & {} \frac{4}{3} [\mathrm{d}\sigma (-1<x<-1/2) \nonumber \\&-\mathrm{d}\sigma (-1/2<x<1/2) +\mathrm{d}\sigma (1/2<x<1) ] \nonumber \\ \end{aligned}$$

(with $x=\cos \theta _1$ or $\cos \theta _2$), is very sensitive to the presence of EFT operators. The $T_2(\cos \theta _2)$ dependence on $\tilde{c}_{HW}$ is presented in Fig. 12 (upper panel) for illustrative purposes. In this example, we observe a $\tilde{c}_{HW}$ dependence that could be exploited by precise measurements. We additionally show, in the lower panel of the figure, the invariant-mass distribution of the $Z_2$ system that additionally feature a dependence on the EFT parameters and could provide an extra handle to better corner deviations from the Standard Model.

## Discussion

We have attempted to find new avenues for probing the impact of possible *CP*-odd interactions of the Higgs boson. We have considered two different approaches. First, we have made use of total rate measurements to both evaluate the current status of the constraints on all bosonic effective *CP*-odd operators and their prospects. Second, we have considered pairs of observables that allows one in principle to get a joint sensitivity to the EFT and CPV effects. One observable is dimensionful so that large momentum transfers could be probed, and another observable involves angles so that the CPV impact is expected to be significant.

We have shown that the constraints that can be derived on the basis of the Run I LHC cross section results will only be barely improved during the next 20 years. Going beyond the total rate approach is thus mandatory in order to corner the Higgs sector better. Differential distributions are powerful handles for a variety of processes. We recast the dimensionful observable as an efficiency of selecting a part of the phase space where the observable under consideration satisfies some condition. On the other hand, the angular observable is connected to an asymmetry.

Our findings can be summarized as follows.

- VH production: The dimensionful observable is taken to be the scalar sum of the transverse momenta of the two leptons originating from the decay of *Z*-boson in the *ZH* case, and the transverse mass of the system comprised of the reconstructed Higgs boson and the lepton issued from the *W*-boson decay in the *WH* case. The angular observable is taken to be the difference in azimuthal angle between the two leptons (the lepton and the missing momentum) in the *ZH* (*WH*) case. We have found that this efficiency and the asymmetry built from the angular observable provide an effective handle to distinguish CPV effects
- VBF production: Similarly, we make use of the azimuthal angular separation of the diphoton system arising from the Higgs-boson decay and the transverse momentum of the leading jet.
- Dileptonic *W*-boson pair production: Here, we use the invariant mass of the dilepton system for computing the efficiency related to the dimensionful observable, and the triple product observable $\tilde{\mathcal {O}}_{1}$ as a dimensionless variable.
- Higgs decays in four-lepton final state: In this case, we rely on the reconstructed off-shell *Z*-boson stemming from the Higgs-boson decay. We consider its invariant mass as a dimensionful variable, and the so-called $T_2$ function applied on the polar angles of its decay products as the dimen sionless variable.

In order to be able to compare the sensitivity expected by the usage of pairs of observables with respect to the use of cross section measurements, there are two ways. Either we need to rely on the corresponding experimental studies, which have not yet been performed, or we need to perform ourselves the simulation of both the signal and the Standard Model background including the parton shower and hadronization effects, as well as the simulation of the impact of the detector response.

**Fig. 13:**
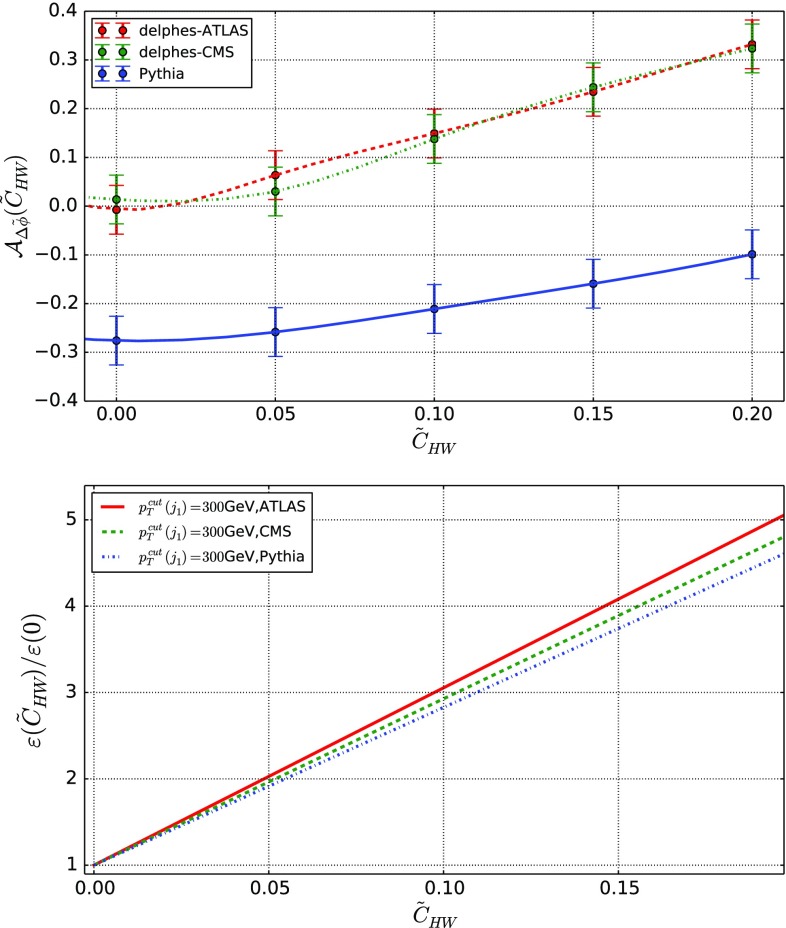
Evaluation of the detector impact on the asymmetry (top) and efficiency (bottom) introduced in the context of VBF Higgs-boson production and defined in Sect. 6.2. We compare predictions solely including parton shower and hadronization effects (blue) to predictions embedding the modeling of the ATLAS (red) and CMS (green) detector effects

As a first step in the second direction, we evaluate in Fig. 13 the effects that could stem from the parton showering and hadronization as modeled by Pythia [90], and those related from the modeling of the ATLAS and CMS detectors as implemented in Delphes [91]. In all cases, object reconstruction is performed by using the anti-$k_T$ jet algorithm [92] as implemented in FastJet [93]. We present results for the two observables introduced in the context of VBF Higgs-boson production in Sect. 6.2. Whereas the $\varepsilon$ efficiency is barely sensitive to detector effects that impact the results by only a few percents, drastic changes are induced in the distribution of the ${\mathcal {A}}_{\varDelta \tilde{\phi }}$ observable. Additionally, we also observe significant changes in the normalization with respect to the parton-level results of Sect. 6.2, but the shape dependence on the Wilson coefficient remains unaltered. It turns out to be even more pronounced when the detector simulation is included, which reinforces the motivation for using this variable to characterize new physics in an EFT context.

The above observables thus require a dedicated study to be performed with the armory of full experimental set up including dedicated high $p_{T}$ triggers and a full data driven background analysis.

## Conclusion

In this paper, we have investigated novel ideas to look for CPV new physics effects arising both in the couplings of the Higgs boson to the weak vector bosons and in the self-interactions of the latter. In order to assess those effects, we have performed an analysis in the context of an effective field theory once the higher-dimensional part of the Lagrangian is restricted to relevant CPV operators. We have studied the impact of these new physics EFT operators on both total rates and differential distributions, as the effects are known to be larger for processes involving large momentum transfer.

We have first used LHC Run I data to define the range in which the considered Wilson coefficients are allowed to vary on the basis of total rate information. We have then explored the prospects for the next runs of the LHC when we restrict the analysis to the usage of similar techniques. The expected improvements have been found rather mild, so that we have investigated how the use of differential information could play a more important role for maximizing the potential of future LHC data.

We have more precisely examined a variety of Higgs and electroweak boson production channels to evaluate the sensitivity of the LHC to new CPV effective operators. Our analysis has included a focus on the associated production of a Higgs and a weak boson (*VH*), Higgs-boson production by vector-boson fusion (VBF), *W*-boson pair production ($W^+W^-$) and the four-lepton channel traditionally used for CPV Higgs-boson studies. In each case, we have studied various kinematic distributions and we have selected the most sensitive ones to EFT effects. We have further proposed several dimensionless (angular) and dimensionful observable that could be used, possibly jointly, as novel handles to pin down new physics.

In this work, we have undertaken, as a pioneering study of these new observables, a beyond the Standard Model signal analysis at the leading-order accuracy in QCD after matching the fixed-order results to parton showers. A more precise assessment on the LHC sensitivity to CPV EFT operators through the use of the new variables that we have proposed, however, necessitates, on the one hand, a full signal and Standard Model background analysis for different luminosity goals and after including the simulation of detector effects. On the other hand, it is also mandatory to evaluate the impact of higher-order corrections to the signal.

The analysis of the background effects and the design of a signal and background analysis is left for future work, assuming that the signals considered in this work are sufficiently distinguishable from the Standard Model (as has so far been the case). Other aspects could also be investigated in the future, like the determination (and disentangling) of possible correlations between *CP*-odd and *CP*-even EFT operator effects in the light of the proposed variables, a statistical combination of all 13 TeV data information possibly merged to experimental low-energy data, as well as the impact on cosmology and more precisely electroweak baryogenesis.

## Appendix A: Event simulation and selection details

In order to simulate all LHC collision events required for this work, we have used as a theoretical context the Standard Model effective field theory expressed in the strongly interacting light Higgs basis [38, 40], also known as the SILH basis. We have made used of the corresponding implementation [42] in the FeynRules package [94] to generate a UFO model [95] that we have used within the MadGraph5_aMC@NLO platform [96]. We have generated, for different choices of the EFT parameters, 150,000 hard scattering events that we have then passed to Pythia 6 [90] for parton showering and hadronization. The final-state objects have been reconstructed by employing the anti-$k_T$ algorithm [92] with an *R*-parameter set to 0.4 by using the FastJet [93] interface of MadAnalysis 5 [97, 98]. The latter program has also been used to achieve all the analyses performed in this work, after considering as *b*-tagged jets all jets for which a *B*-hadron is present within a cone of radius $R=0.4$ centered on the jet momentum direction.

### A.1: *ZH* associated production in the dilepton channel

Reconstructed events are selected by demanding the presence of two isolated leptons whose pseudorapidity satisfies $|\eta |<2.5$ and transverse momentum $p_T$ is larger than 20 GeV. Moreover, we impose the requirement that the invariant mass of the dilepton system is compatible with a *Z*-boson $m_{\ell \ell } \in [83, 110]$ GeV. Lepton isolation is implemented by forbidding the presence of any reconstructed object in a cone of radius $R = 0.4$ centered on the lepton direction. We additionally require that the selected events feature two *b*-tagged jets with a pseudorapidity $|\eta |<2.5$ and a transverse momentum larger than 40 and 20 GeV for the leading and subleading *b*-jet, respectively.

### A.2: *WH* associated production in the single-lepton channel

We select events whose particle content features a single isolated charged lepton with a transverse momentum $p_T>10$ GeV and a pseudorapidity $|\eta |< 2.47$, two *b*-tagged jets with a transverse momentum greater than 40 and 20 GeV for the leading and subleading jet, respectively, and with a pseudorapidity $|\eta |<2.5$. Lepton isolation is implemented by forbidding the presence of any reconstructed object in a cone of radius $R = 0.4$ centered on the lepton direction.

### A.3: VBF Higgs-boson production

Events are selected by requiring the presence of two jets with a transverse momentum $p_{T}^{j}>20$ GeV, a pseudrapidity $|\eta _{j}|<4.5$ and typical VBF properties. The dijet invariant mass hence required to be larger than 400 GeV and the jet separation in pseudrapidity is imposed to be above 2.8.

### A.4: *W*-boson pair production

We select events featuring a final state with two isolated leptons whose pseudorapidity satisfies $|\eta |<2.5$ and with a transverse momentum larger than 20 GeV. Lepton isolation is implemented by forbidding the presence of any reconstructed object lying in a cone of radius $R = 0.4$ centered on the lepton direction, the jet candidate being jets with a transverse momentum larger than 20 GeV and a pseudorapidity smaller than 4.5 in absolute value.

### A.5: Higgs-boson production and decay into the four-lepton channel

Event selection relies on the presence of four isolated leptons with a pseudorapidity $|\eta |<2.5$ and a transverse momentum $p_T>10$ GeV in the final state. Jets candidate are defined with a transverse momentum enforced to be larger than 20 GeV and a pseudorapidity smaller than 4.5 in absolute value, and lepton isolation is imposed by forbidding the presence of objects in a cone of radius $R = 0.4$ centered on the lepton direction.

## References

[1] ATLAS Collaboration, G. Aad et al., Observation of a new particle in the search for the Standard Model Higgs boson with the ATLAS detector at the LHC. Phys. Lett. B **716**, 1–29 (2012). arXiv:1207.7214

[2] CMS Collaboration, S. Chatrchyan et al., Observation of a new boson at a mass of 125 GeV with the CMS experiment at the LHC. Phys. Lett. B **716**, 30–61 (2012). arXiv:1207.7235

[3] ATLAS, CMS Collaboration, G. Aad et al., Measurements of the Higgs boson production and decay rates and constraints on its couplings from a combined ATLAS and CMS analysis of the LHC pp collision data at $$ \sqrt{s}=7 $$ and 8 TeV. JHEP **08**, 045 (2016). arXiv:1606.02266

[4] Espinosa JR, Gripaios B, Konstandin T, Riva F (2012). Electroweak baryogenesis in non-minimal composite Higgs models. JCAP.

[5] Corbett T, Eboli OJP, Gonzalez-Fraile J, Gonzalez-Garcia MC (2013). Robust determination of the Higgs couplings: power to the data. Phys. Rev. D.

[6] Dumont B, Fichet S, von Gersdorff G (2013). A Bayesian view of the Higgs sector with higher dimensional operators. JHEP.

[7] de Blas J, Ciuchini M, Franco E, Ghosh D, Mishima S, Pierini M, Reina L, Silvestrini L (2016). Global bayesian analysis of the Higgs-boson couplings. Nucl. Part. Phys. Proc..

[8] Falkowski A, Riva F (2015). Model-independent precision constraints on dimension-6 operators. JHEP.

[9] B. Dumont, Higgs, supersymmetry and dark matter after Run I of the LHC. Ph.D. thesis, LPSC, Grenoble (2014). arXiv:1411.3465

[10] Ellis J, Sanz V, You T (2015). The effective Standard Model after LHC Run I. JHEP.

[11] Ellis J, Sanz V, You T (2014). Complete Higgs sector constraints on dimension-6 operators. JHEP.

[12] Butter A, Éboli OJP, Gonzalez-Fraile J, Gonzalez-Garcia MC, Plehn T, Rauch M (2016). The gauge-Higgs legacy of the LHC Run I. JHEP.

[13] S. Dawson et al., Working group report: Higgs boson, in *Proceedings, 2013 Community Summer Study on the Future of U.S. Particle Physics: Snowmass on the Mississippi (CSS2013): Minneapolis, MN, USA, July 29–August 6, 2013* (2013). arXiv:1310.8361

[14] Anderson I (2014). Constraining anomalous HVV interactions at proton and lepton colliders. Phys. Rev. D.

[15] Delaunay C, Perez G, de Sandes H, Skiba W (2014). Higgs up-down CP asymmetry at the LHC. Phys. Rev. D.

[16] Dwivedi S, Ghosh DK, Mukhopadhyaya B, Shivaji A (2015). Constraints on CP-violating gauge-Higgs operators. Phys. Rev. D.

[17] Gritsan AV, Röntsch R, Schulze M, Xiao M (2016). Constraining anomalous Higgs boson couplings to the heavy flavor fermions using matrix element techniques. Phys. Rev. D.

[18] Khatibi S, Mohammadi Najafabadi M (2014). Exploring the anomalous Higgs-top couplings. Phys. Rev. D.

[19] Dwivedi S, Ghosh DK, Mukhopadhyaya B, Shivaji A (2016). Distinguishing $$CP$$-odd couplings of the Higgs boson to weak boson pairs. Phys. Rev. D.

[20] CMS Collaboration, V. Khachatryan et al., Constraints on the spin-parity and anomalous HVV couplings of the Higgs boson in proton collisions at 7 and 8 TeV. Phys. Rev. D **92**(1), 012004 (2015). arXiv:1411.3441

[21] CMS Collaboration, V. Khachatryan et al., Combined search for anomalous pseudoscalar HVV couplings in VH(H $$\rightarrow b \bar{b}$$) production and H $$\rightarrow $$ VV decay. Phys. Lett. B **759**, 672–696 (2016). arXiv:1602.04305

[22] Weinberg S (1989). Larger Higgs exchange terms in the neutron electric dipole moment. Phys. Rev. Lett..

[23] Particle Data Group Collaboration, S. Eidelman, Gauge and Higgs boson summary table. Phys. Lett. B **592**, 31–88 (2004)

[24] Chien YT, Cirigliano V, Dekens W, de Vries J, Mereghetti E (2016). Direct and indirect constraints on CP-violating Higgs-quark and Higgs-gluon interactions. JHEP.

[25] CMS Collaboration, S. Chatrchyan et al., Study of the mass and spin-parity of the higgs boson candidate via its decays to Z boson pairs. Phys. Rev. Lett. **110**(8), 081803 (2013). arXiv:1212.6639 10.1103/PhysRevLett.110.081803 23473131

[26] Manohar AV, Wise MB (2006). Modifications to the properties of the Higgs boson. Phys. Lett. B.

[27] Chang W-F, Pan W-P, Xu F (2013). Effective gauge-Higgs operators analysis of new physics associated with the Higgs boson. Phys. Rev. D.

[28] H. Belusca-Maito, Effective Higgs Lagrangian and constraints on Higgs couplings. arXiv:1404.5343

[29] H. Belusca-Maito, Higgs couplings in an effective theory framework (2015). arXiv:1507.05657

[30] Choi SY, Miller DJ, Muhlleitner MM, Zerwas PM (2003). Identifying the Higgs spin and parity in decays to Z pairs. Phys. Lett. B.

[31] Godbole RM, Miller DJ, Muhlleitner MM (2007). Aspects of CP violation in the H ZZ coupling at the LHC. JHEP.

[32] Hagiwara K, Li Q, Mawatari K (2009). Jet angular correlation in vector-boson fusion processes at hadron colliders. JHEP.

[33] Englert C, Hackstein C, Spannowsky M (2010). Measuring spin and CP from semi-hadronic ZZ decays using jet substructure. Phys. Rev. D.

[34] LHC Higgs Cross Section Working Group Collaboration, D. de Florian et al., Handbook of LHC Higgs cross sections: 4. Deciphering the nature of the Higgs sector. arXiv:1610.07922

[35] Burges CJC, Schnitzer HJ (1983). Virtual effects of excited quarks as probes of a possible new hadronic mass scale. Nucl. Phys. B.

[36] Leung CN, Love ST, Rao S (1986). Low-energy manifestations of a new interaction scale: operator analysis. Z. Phys. C.

[37] Buchmuller W, Wyler D (1986). Effective Lagrangian analysis of new interactions and flavor conservation. Nucl. Phys. B.

[38] Giudice GF, Grojean C, Pomarol A, Rattazzi R (2007). The strongly-interacting light Higgs. JHEP.

[39] Grzadkowski B, Iskrzynski M, Misiak M, Rosiek J (2010). Dimension-six terms in the Standard Model Lagrangian. JHEP.

[40] Contino R, Ghezzi M, Grojean C, Muhlleitner M, Spira M (2013). Effective Lagrangian for a light Higgs-like scalar. JHEP.

[41] Gupta R S, Pomarol A, Riva F (2015). BSM primary effects. Phys. Rev. D.

[42] Alloul A, Fuks B, Sanz V (2014). Phenomenology of the Higgs effective Lagrangian via FEYNRULES. JHEP.

[43] Alonso R, Jenkins EE, Manohar AV, Trott M (2014). Renormalization group evolution of the Standard Model dimension six operators III: gauge coupling dependence and phenomenology. JHEP.

[44] Falkowski A, Fuks B, Mawatari K, Mimasu K, Riva F, sanz V (2015). Rosetta: an operator basis translator for Standard Model effective field theory. Eur. Phys. J. C.

[45] Berge S, Bernreuther W, Ziethe J (2008). Determining the CP parity of Higgs bosons at the LHC in their tau decay channels. Phys. Rev. Lett..

[46] Berge S, Bernreuther W (2009). Determining the CP parity of Higgs bosons at the LHC in the tau to 1-prong decay channels. Phys. Lett. B.

[47] Berge S, Bernreuther W, Niepelt B, Spiesberger H (2011). How to pin down the CP quantum numbers of a Higgs boson in its tau decays at the LHC. Phys. Rev. D.

[48] Brod J, Haisch U, Zupan J (2013). Constraints on CP-violating Higgs couplings to the third generation. JHEP.

[49] Harnik R, Martin A, Okui T, Primulando R, Yu F (2013). Measuring CP violation in $$h \rightarrow \tau ^+ \tau ^-$$ at colliders. Phys. Rev. D.

[50] Henning B, Lu X, Murayama H (2016). How to use the Standard Model effective field theory. JHEP.

[51] Gorbahn M, No JM, Sanz V (2015). Benchmarks for Higgs effective theory: extended Higgs sectors. JHEP.

[52] Drozd A, Ellis J, Quevillon J, You T (2015). Comparing EFT and exact one-loop analyses of non-degenerate stops. JHEP.

[53] C. Degrande, B. Fuks, K. Mawatari, K. Mimasu, V. Sanz, Electroweak Higgs boson production in the standard model effective field theory beyond leading order in QCD. Eur. Phys. J. **C77**(4), 262 (2017). arXiv:1609.04833

[54] Contino R, Falkowski A, Goertz F, Grojean C, Riva F (2016). On the validity of the effective field theory approach to SM precision tests. JHEP.

[55] Gripaios B, Pomarol A, Riva F, Serra J (2009). Beyond the minimal composite Higgs model. JHEP.

[56] Sanz V, Setford J (2015). Composite Higgses with seesaw EWSB. JHEP.

[57] Galloway J, Evans JA, Luty MA, Tacchi RA (2010). Minimal conformal technicolor and precision electroweak tests. JHEP.

[58] Croon D, Sanz V, Tarrant E R M (2016). Reheating with a composite Higgs boson. Phys. Rev. D.

[59] No J M, Sanz V, Setford J (2016). See-saw composite Higgs model at the LHC: linking naturalness to the 750 GeV diphoton resonance. Phys. Rev. D.

[60] Carena M, Ellis JR, Pilaftsis A, Wagner CEM (2000). CP violating MSSM Higgs bosons in the light of LEP-2. Phys. Lett. B.

[61] Carena M, Ellis JR, Pilaftsis A, Wagner CEM (2002). Higgs boson pole masses in the MSSM with explicit CP violation. Nucl. Phys. B.

[62] Carena M, Ellis JR, Mrenna S, Pilaftsis A, Wagner CEM (2003). Collider probes of the MSSM Higgs sector with explicit CP violation. Nucl. Phys. B.

[63] Ellis JR, Lee JS, Pilaftsis A (2007). B-Meson observables in the maximally CP-violating MSSM with minimal flavour violation. Phys. Rev. D.

[64] Carena M, Ellis J, Lee JS, Pilaftsis A, Wagner CEM (2016). CP violation in heavy MSSM Higgs scenarios. JHEP.

[65] Ellis J, Hwang DS, Sanz V, You T (2012). A fast track towards the ‘Higgs’ spin and parity. JHEP.

[66] Ellis J, Sanz V, You T (2013). Associated production evidence against Higgs impostors and anomalous couplings. Eur. Phys. J. C.

[67] ATLAS Collaboration, M. Aaboud et al., Measurement of $$W^+W^-$$ production in association with one jet in proton–proton collisions at $$\sqrt{s} =8$$ TeV with the ATLAS detector. Phys. Lett. B **763**, 114–133 (2016). arXiv:1608.03086

[68] Campbell JM, Ellis RK, Williams C (2011). Vector boson pair production at the LHC. JHEP.

[69] LHC Higgs Cross Section Working Group Collaboration, J.R. Andersen et al., Handbook of LHC Higgs cross sections: 3. Higgs properties. arXiv:1307.1347

[70] Gehrmann T, Grazzini M, Kallweit S, Maierhöfer P, von Manteuffel A, Pozzorini S, Rathlev D, Tancredi L (2014). $$W^+W^-$$ production at hadron colliders in next to next to leading order QCD. Phys. Rev. Lett..

[71] ATLAS Collaboration, G. Aad et al., Measurement of total and differential $$W^+W^-$$ production cross sections in proton-proton collisions at $$\sqrt{s}=$$ 8 TeV with the ATLAS detector and limits on anomalous triple-gauge-boson couplings. JHEP **09**, 029 (2016). arXiv:1603.01702

[72] ATLAS Collaboration, Projections for measurements of Higgs boson signal strengths and coupling parameters with the ATLAS detector at a HL-LHC. ATL-PHYS-PUB-2014-016 (2014)

[73] ATLAS Collaboration, Update of the prospects for the $$H \rightarrow Z\gamma $$ search at the high-luminosity LHC. ATL-PHYS-PUB-2014-006 (2014)

[74] Grazzini M, Kallweit S, Pozzorini S, Rathlev D, Wiesemann M (2016). $$W^+W^-$$ production at the LHC: fiducial cross sections and distributions in NNLO QCD. JHEP.

[75] Ellis J, Sanz V, You T (2013). Prima facie evidence against spin-two Higgs impostors. Phys. Lett. B.

[76] Han T, Li Y (2010). Genuine CP-odd observables at the LHC. Phys. Lett. B.

[77] Christensen ND, Han T, Li Y (2010). Testing CP violation in ZZH interactions at the LHC. Phys. Lett. B.

[78] E. Fuchs, G. Weiglein, Impact of CP-violating interference effects on MSSM Higgs searches. arXiv:1705.05757

[79] Fowler AC, Weiglein G (2010). Precise predictions for Higgs production in neutralino decays in the complex MSSM. JHEP.

[80] Hankele V, Klamke G, Zeppenfeld D, Figy T (2006). Anomalous Higgs boson couplings in vector boson fusion at the CERN LHC. Phys. Rev. D.

[81] Belyaev N, Konoplich R, Pedersen L E, Prokofiev K (2015). Angular asymmetries as a probe for anomalous contributions to HZZ vertex at the LHC. Phys. Rev. D.

[82] Plehn T, Rainwater DL, Zeppenfeld D (2002). Determining the structure of Higgs couplings at the LHC. Phys. Rev. Lett..

[83] Desai N, Ghosh DK, Mukhopadhyaya B (2011). CP-violating HWW couplings at the large hadron collider. Phys. Rev. D.

[84] Stolarski D, Vega-Morales R (2012). Directly measuring the tensor structure of the scalar coupling to gauge bosons. Phys. Rev. D.

[85] Freitas A, Schwaller P (2013). Higgs CP properties from early LHC data. Phys. Rev. D.

[86] Chen Y, Vega-Morales R (2014). Extracting effective Higgs couplings in the golden channel. JHEP.

[87] Bishara F, Grossman Y, Harnik R, Robinson DJ, Shu J, Zupan J (2014). Probing CP violation in $$h\rightarrow \gamma \gamma $$ with converted photons. JHEP.

[88] Chen Y, Di Marco E, Lykken J, Spiropulu M, Vega-Morales R, Xie S (2015). 8D likelihood effective Higgs couplings extraction framework in $$h \rightarrow 4\ell $$. JHEP.

[89] Chen Y, Falkowski A, Low I, Vega-Morales R (2014). New observables for CP violation in Higgs decays. Phys. Rev. D.

[90] Sjostrand T, Mrenna S, Skands P Z (2006). PYTHIA 6.4 physics and manual. JHEP.

[91] DELPHES 3 Collaboration, J. de Favereau, C. Delaere, P. Demin, A. Giammanco, V. Lemaître, A. Mertens, M. Selvaggi, DELPHES 3, A modular framework for fast simulation of a generic collider experiment. JHEP **02**, 057 (2014). arXiv:1307.6346

[92] Cacciari M, Salam GP, Soyez G (2008). The anti-k(t) jet clustering algorithm. JHEP.

[93] Cacciari M, Salam GP, Soyez G (2012). FastJet user manual. Eur. Phys. J. C.

[94] Alloul A, Christensen ND, Degrande C, Duhr C, Fuks B (2014). FeynRules 2.0—a complete toolbox for tree-level phenomenology. Comput. Phys. Commun..

[95] Degrande C, Duhr C, Fuks B, Grellscheid D, Mattelaer O, Reiter T (2012). UFO—the universal FeynRules output. Comput. Phys. Commun..

[96] Alwall J, Frederix R, Frixione S, Hirschi V, Maltoni F, Mattelaer O, Shao HS, Stelzer T, Torrielli P, Zaro M (2014). The automated computation of tree-level and next-to-leading order differential cross sections, and their matching to parton shower simulations. JHEP.

[97] Conte E, Fuks B, Serret G (2013). MadAnalysis 5, a user-friendly framework for collider phenomenology. Comput. Phys. Commun..

[98] Conte E, Dumont B, Fuks B, Wymant C (2014). Designing and recasting LHC analyses with MadAnalysis 5. Eur. Phys. J. C.

